# Aging rather than aneuploidy affects monoamine neurotransmitters in brain regions of Down syndrome mouse models

**DOI:** 10.1016/j.nbd.2017.06.007

**Published:** 2017-09

**Authors:** Alain D. Dekker, Yannick Vermeiren, Christelle Albac, Eva Lana-Elola, Sheona Watson-Scales, Dorota Gibbins, Tony Aerts, Debby Van Dam, Elizabeth M.C. Fisher, Victor L.J. Tybulewicz, Marie-Claude Potier, Peter P. De Deyn

**Affiliations:** a Department of Neurology and Alzheimer Research Center, University of Groningen, University Medical Center Groningen, Hanzeplein 1, 9713 GZ Groningen, The Netherlands; b Laboratory of Neurochemistry and Behaviour, Institute Born-Bunge, University of Antwerp, Universiteitsplein 1, 2610 Wilrijk, Antwerp, Belgium; c Sorbonne Universités, UPMC Université Paris 06, INSERM, CNRS (U75, U1127, U7225) and Institut du Cerveau et de la Moelle Epinière (ICM), Boulevard de l'Hôpital, 75013 Paris, France; d The Francis Crick Institute, Midland Road, London NW1 1AT, United Kingdom; e Department of Neurodegenerative Disease, Institute of Neurology, University College London, Queen Square, London WC1N 3BG, United Kingdom; f Department of Medicine, Imperial College London, Du Cane Road, London W12 0NN, United Kingdom

**Keywords:** Aging, Dopamine, Down syndrome, Dp1Tyb, Monoamines, Mouse models, Noradrenaline, RP-HPLC, Serotonin, Ts65Dn

## Abstract

Altered concentrations of monoamine neurotransmitters and metabolites have been repeatedly found in people with Down syndrome (DS, trisomy 21). Because of the limited availability of human post-mortem tissue, DS mouse models are of great interest to study these changes and the underlying neurobiological mechanisms. Although previous studies have shown the potential of Ts65Dn mice – the most widely used mouse model of DS – to model noradrenergic changes, a comprehensive monoaminergic characterization in multiple brain regions has not been performed so far. Here, we used RP-HPLC with electrochemical detection to quantify (nor)adrenergic (NA, adrenaline and MHPG), dopaminergic (DA, HVA and DOPAC), and serotonergic compounds (tryptophan, 5-HT and 5-HIAA) in ten regionally dissected brain regions of Ts65Dn mice, as well as in Dp1Tyb mice – a novel DS mouse model. Comparing young adult aneuploid mice (2.5–5.5 months) with their euploid WT littermates did not reveal generalized monoaminergic dysregulation, indicating that the genetic overload in these mice barely affected the absolute concentrations at this age. Moreover, we studied the effect of aging in Ts65Dn mice: comparing aged animals (12–13 months) with their younger counterparts revealed a large number of significant changes. In general, the (nor)adrenergic system appeared to be reduced, while serotonergic compounds were increased with aging. Dopaminergic alterations were less consistent. These overall patterns appeared to be relatively similar for Ts65Dn and WT mice, though more observed changes were regarded significant for WT mice. Similar human post-mortem studies are necessary to validate the monoaminergic construct validity of the Ts65Dn and Dp1Typ mouse models.

## Highlights

• Monoamine neurotransmitters and metabolites appear to be altered in Down syndrome.
• The monoaminergic brain profile of two Down syndrome mouse models was examined.
• Aneuploidy barely affected monoamines in Ts65Dn and Dp1Tyb mice vs. wild-type mice.
• Aging to 12–13 months showed strong monoaminergic changes in the Ts65Dn mouse model.
• Construct validity needs to be established by similar human post-mortem studies.

## 1 Introduction

Down syndrome (DS), caused by an additional copy of chromosome 21 (HSA21), is the most common intellectual disability with a genetic origin affecting nearly six million people worldwide (Ballard et al., 2016). DS is generally characterized by behavioral alterations (Dekker et al., 2015b) and reduced cognitive capacities, in particular impaired verbal short-term memory, explicit long-term memory, morphosyntax and an average IQ of 45 (Lott and Dierssen, 2010, Vicari et al., 2004). Moreover, people with DS are at an extremely high risk to develop dementia: 68–80% develop Alzheimer's disease (AD) by the age of 65 years (Wiseman et al., 2015), compared to 11% in the general (non-intellectually disabled) population of 65 years and older (Alzheimer's Assocation, 2016).

**Fig. 1:**
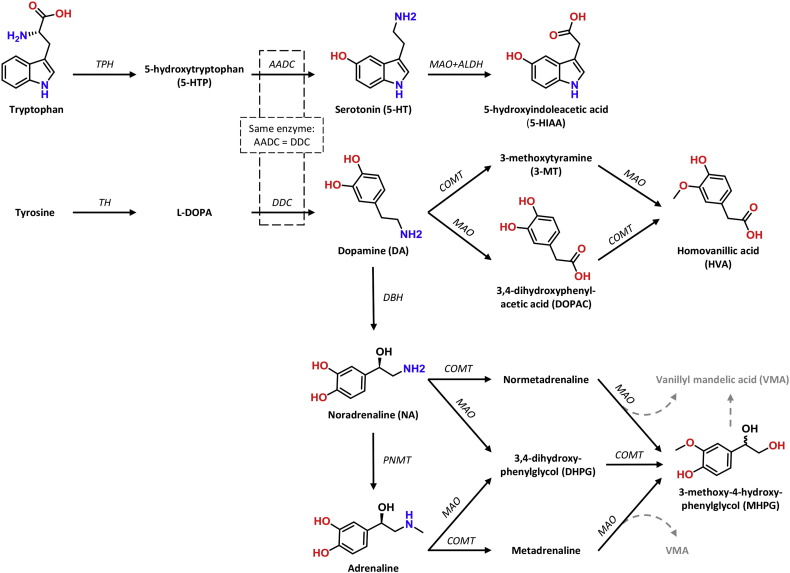
Schematic biosynthesis routes of monoamine neurotransmitters and their main metabolites. 5-HT is derived from the amino acid tryptophan, whereas DA (and thus NA and adrenaline) are derived from the amino acid tyrosine. The molecular structures are provided for the compounds that are quantified in this study through reversed-phase high-performance liquid chromatography (RP-HPLC) analyses. Abbreviations: AADC, aromatic amino acid decarboxylase; ALDH, aldehyde dehydrogenase; COMT, catechol-*O*-methyltransferase; DBH, dopamine β-hydroxylase; DDC, DOPA decarboxylase; l-DOPA, l-3,4-dihydroxyphenylalanine (levodopa); MAO, monoamine oxidase; PNMT, phenylethanolamine *N*-methyltransferase; TH, tyrosine hydroxylase; TPH, tryptophan hydroxylase.

Elucidating the underlying neurobiological mechanisms affecting behavior and cognition in DS would greatly contribute to understanding the pathophysiology, as well as facilitate development of novel disease-modifying strategies. Of particular interest in this population are monoamine neurotransmitters: noradrenaline (NA), adrenaline, dopamine (DA) and serotonin (5-HT) and their metabolites (summarized in Fig. 1). A series of neurochemical studies has shown significant alterations in one or more monoamines in various brain regions (Godridge et al., 1987, Reynolds and Godridge, 1985, Risser et al., 1997, Whittle et al., 2007, Yates et al., 1981), cerebrospinal fluid (CSF) (Kay et al., 1987, Schapiro et al., 1987) and serum/plasma (Coppus et al., 2007, Dekker et al., 2015a) of DS individuals as compared to non-DS controls.

Although monoaminergic alterations in CSF and serum/plasma likely reflect changes in the brain, direct analysis of brain tissue is warranted to understand central neurotransmission alterations. Indeed, we previously studied monoamines and metabolites in post-mortem brain regions of behaviorally characterized patients in the general population with (early-onset) AD (Vermeiren et al., 2014a, Vermeiren et al., 2014b, Vermeiren et al., 2015, Vermeiren et al., 2016), frontotemporal dementia (Vermeiren et al., 2016) and dementia with Lewy bodies (Vermeiren et al., 2015). Surprisingly, despite the high risk for dementia in DS, only a few post-mortem studies dating back to the 1980s have investigated monoamines in DS. Unfortunately, post-mortem brain samples from clinically and pathologically well-documented DS individuals have been rarely included in biobanks around the world, severely limiting the possibilities for such research.

Thus, valid mouse models of DS are of great interest to study the potential monoaminergic alterations related to trisomy 21. In addition to the differences between DS and non-DS controls, monoaminergic changes have been reported in DS mouse models, in particular noradrenergic alterations (Dierssen et al., 1997, Lockrow et al., 2011, Salehi et al., 2009). These findings have been primarily observed in Ts(17^16^)65Dn mice (Ts65Dn in short), the most widely used and best characterized DS model. HSA21 contains 233 protein-encoding genes: among those genes with a homologue in mice, the majority (~ 58%) is found on a large segment of mouse chromosome 16 (Mmu16), and to a lesser extent on shorter segments of Mmu10 and Mmu17 (Lana-Elola et al., 2016). Ts65Dn mice carry an additional mini-chromosome that is formed by the translocation of a duplicated segment of Mmu16 to a small part of Mmu17 (Davisson et al., 1990), making them trisomic for approximately 50% of the genes homologous to HSA21, but also for 60 non-homologous genes on Mmu17 (Duchon et al., 2011).

Of profound interest is the significant loss of neurons in the locus coeruleus (LC), the key production site of NA in the pons, in Ts65Dn mice of 12 months of age but not in Ts65Dn mice at 4 months of age as compared to age-matched euploid wildtype (WT_Ts65Dn_) littermates (Fortress et al., 2015, Lockrow et al., 2011). Neurodegeneration with progressive aging was further demonstrated by the significant loss of axonal processes and shrinkage of the noradrenergic neurons between 4 and 12 months in Ts65Dn, but not in WT_Ts65Dn_ (Lockrow et al., 2011). Similarly, Salehi and colleagues found a reduced number of LC neurons in Ts65Dn mice at 6 months and 18 months of age, but not in those of 3 months of age, as compared to age-matched WT_Ts65Dn_ mice. Moreover, NA levels in the hippocampus were significantly lower in Ts65Dn than in WT_Ts65Dn_ at 18 months. Despite the pronounced LC degeneration, impaired NA-modulated contextual learning could be rescued in these mice after treatment with the noradrenergic prodrug l-threo-3,4-dihydroxyphenylserine (l-DOPS) or the β1-adrenergic receptor partial agonist xamoterol (Salehi et al., 2009). Restoration of impaired noradrenergic neurotransmission may thus serve as potential disease-modifying therapy in DS (Phillips et al., 2016).

Despite a large number of studies using Ts65Dn mice, a comprehensive monoaminergic characterization of the brain of this mouse model has not been conducted so far. Therefore, this study aimed to investigate the potential of DS mouse models (construct validity) in modelling the monoaminergic changes in DS by (1) establishing the monoaminergic profile in ten regionally dissected brain regions of aneuploid Ts65Dn mice in comparison to their euploid WT_Ts65Dn_ littermates, and (2) studying the effect of aging in Ts65Dn and WT_Ts65Dn_ mice. Using an optimized reversed-phase high-performance liquid chromatography (RP-HPLC) set-up with electrochemical detection we detected and quantified (nor)adrenaline and the metabolite 3-methoxy-4-hydroxyphenylglycol (MHPG), DA with its metabolites homovanillic acid (HVA) and 3,4-dihydroxyphenylacetic acid (DOPAC), tryptophan, and 5-HT with its metabolite 5-hydroxyindoleacetic acid (5-HIAA) in each of these brain regions.

Whereas Ts65Dn mice have proven very useful for studying various phenotypic features present in people with DS (Gardiner, 2015), the additional 60 genes from Mmu17 that are not homologous to HSA21 may complicate the interpretation of generated data (Duchon et al., 2011). Therefore, new mouse models have been developed that are only trisomic for genes homologous to HSA21, such as the Dp(16)1Yey strain that is trisomic for the entire homologous segment on Mmu16 (Yu et al., 2010). Similar to Dp(16)1Yey, the Dp1Tyb mouse model has been generated to exclude the effect of the irrelevant Mmu17 genes triplicated in Ts65Dn. Using Cre/loxP-mediated recombination, Dp1Tyb mice were engineered with a triplication of Mmu16 genes from *Lipi* to *Zbtb21* including 148 protein-encoding Mmu16 genes (Lana-Elola et al., 2016). Given the debate on the non-homologous genes in Ts65Dn mice, we also performed a first exploratory analysis of the monoaminergic profile of this new Dp1Typ mouse model.

## 2 Materials & methods

### 2.1 Animals

As Ts65Dn is the most widely used DS mouse model, the aim of this project was to establish its monoaminergic profile and evaluate the effect of aging in this model. Since Ts65Dn are trisomic for 60 Mmu17 genes that are not homologous to HSA21, we also conducted a first (pilot) study with young Dp1Tyb mice, which resemble the human situation more closely from a genetic point of view. Given the time-intensive nature of this project, the availability of aged Ts65Dn mice and that the aforementioned studies in Ts65Dn implicated monoaminergic changes with aging, possibly resembling the changes in humans, we studied the effect of aging only in the Ts65Dn mouse model.

#### 2.1.1 Ts65Dn mice

Male Ts65Dn (n = 32) and euploid WT_Ts65Dn_ littermates (n = 28) were bred, aged and sacrificed in the Institut du Cerveau et de la Moelle Epinière (ICM, Paris, France). To study the effect of aging, we considered two age ranges (Table 1): young adult animals (~ 3–5.5 months) and aged animals (~ 12–13 months). Since the original homozygous Ts65Dn mice suffer from blindness due to the recessive retinal degeneration 1 mutation Pde6b^rd1^, we used an alternative strain that is wild-type for Pde6b^rd1^ (The Jackson Laboratory, Bar Harbor, USA, stock number 005252), thus preventing retinal degeneration. The Ts65Dn colony was maintained by crossing trisomic Ts65Dn females (B6EiC3Sn.BLiA-Ts(17^16^)65Dn/DnJ) to B6EiC3Sn.Bli males (The Jackson Laboratory, stock numbers 005252 and 003647, respectively). Mixed-genotype groups were housed under standard conditions with ad libitum food and water, constant room temperature and a 12 h light/dark cycle. The experiments were compliant with the ethical standards and animal welfare regulations of the French Ministry of Agriculture and the EU Directive 2010/63/EU. MC Potier has the authorization for experiments on vertebrates (N°A-75-2138, Direction Départementale de la Protection des Populations de Paris, Service Protection et Santé Animales, Environnement).

**Table 1:** Overview of experimental groups.

Experimental groups	Number of mice	Age in days
Ts65Dn-young	21	117 (91–174)
WT_Ts65Dn_-young	18	118.5 (110–174)
Ts65Dn-aged	11	377 (354–399)
WT_Ts65Dn_-aged	10	382 (376–382)
Dp1Tyb	9	82 (79–93)
WT_Dp1Tyb_	9	84 (79–93)

Age in days is provided as median with the age range between brackets.

#### 2.1.2 Dp1Tyb mice

The engineering of the C57BL/6J.129P2-Dp(16Lipi-Zbtb21)1TybEmcf/Nimr (Dp1Tyb) strain is described in Lana-Elola et al. (2016). In this first exploratory analysis of this model, we used young male Dp1Tyb (n = 9) mice and euploid WT_Dp1Tyb_ littermates (n = 9) aged 2.5–3 months (Table 1). All mice had been backcrossed at least 5 generations onto the C57BL/6J background. The animals were bred, aged and sacrificed in the Francis Crick Institute (London, UK). Animals were housed in specific pathogen free conditions, with ad libitum food and water, constant room temperature and a 12 h light/dark cycle. The characterization and breeding of Dp1Tyb mice was carried out under a Project License granted by the UK Home Office, and in accordance with the EU Directive 2010/63/EU.

### 2.2 Sacrifice

After intraperitoneal anesthesia with pentobarbital, mice were sacrificed by cervical dislocation and subsequently fully submersed in liquid nitrogen (3 min) to stop metabolization of monoamines. Mice were stored at − 80 °C and shipped on dry ice to the Laboratory of Neurochemistry and Behaviour in Antwerp for further processing.

### 2.3 Regional brain dissection

Brains were extracted from the frozen mice using surgical tools, and subsequently micro-dissected on a cold plate under a binocular microscope, resembling a previously published protocol (Van Dam et al., 2005). Per mouse, ten (sub)cortical brain regions were obtained: frontal cortex, temporal cortex, parietal cortex, occipital cortex, hippocampus, striatum, (hypo)thalamus, brainstem, cerebellum and olfactory bulb. The weight of the brain samples was determined in pre-weighted Eppendorf tubes. Samples were kept at − 80 °C until RP-HPLC analyses.

### 2.4 Sample preparation

Brain samples were defrosted to 4 °C and subsequently homogenized in 800 μl sample buffer (50 mM citric acid, 50 mM phosphoric acid, 0.1 mM EDTA, 8 mM KCl and 1.8 mM octane-1-sulfonic acid sodium salt (OSA), adjusted to pH = 3.6) using a Bio-Gen PRO200 homogenizer (PRO Scientific Inc., Oxford, CT, USA). Thereupon, 450 μl homogenate was transferred onto a 3000 Da Amicon® Ultra 0.5 Centrifugal Filter (Millipore, Ireland) that had been pre-washed (2 ×) using 450 μl sample buffer (centrifugation: 14,000 ×* g*, 25 min, 4 °C). The Amicon® filter loaded with homogenate was centrifuged (14,000 ×* g*, 40 min, 4 °C). Per region per mouse, 5 μl of the resulting filtrate was simultaneously injected by an Alexys™ AS 100 automatic sample injector onto two parallel ALF-125 columns.

### 2.5 RP-HPLC

Noradrenergic (NA, adrenaline and MHPG), dopaminergic (DA, DOPAC and HVA) and serotonergic compounds (TRP, 5-HT and 5-HIAA) were simultaneously quantified in one run using an optimized RP-HPLC set-up with ion-pairing (OSA) and amperometric electrochemical detection, as published before (Dekker et al., 2015a, Van Dam et al., 2014). In summary, we used an Alexys™ Dual Monoamines Analyzer (Antec Leyden BV, Zoeterwoude, The Netherlands), including two LC 110 pumps for a constant flow rate (40 μl/min), two parallel microbore ALF-125 columns (250 mm × 1 mm, 3 μm particle size, 36 °C) with a porous C_18_ silica stationary phase for separation of the compounds and two Decade II electrochemical amperometric detectors with VT03 electrochemical flow cells containing an In Situ Ag/AgCl reference electrode (ISAAC) at 670 mV and a 0.7 mm glassy carbon working electrode. RP-HPLC runs were conducted per brain region and compounds were analyzed in duplicate (output ranges resp. 1 nA and 500 pA). The mobile phase consisted of the aforementioned sample buffer with 13% methanol as organic modifier. Dihydroxybenzylamine hydrochloride (DHBA) and 5-hydroxy-*N*-methyl tryptamine oxalate (5-HMT) were used as internal standards. Chromatograph analyses were performed with Clarity™ software (DataApex Ltd., Prague, Czech Republic).

### 2.6 Statistics

Histograms and Shapiro-Wilk tests (P < 0.05) demonstrated that the monoamines and metabolites did not have a normal distribution overall. Accordingly, non-parametric tests were selected: Mann Whitney *U* tests were used to compare all monoaminergic data between Dp1Tyb and WT_Dp1Tyb_ littermates, whereas Kruskal-Wallis with post-hoc Mann Whitney *U* tests were applied to compare the young Ts65Dn, young WT_Ts65Dn_, aged Ts65Dn and aged WT_Ts65Dn_ groups. To account for multiple comparisons we corrected the P-values using the Benjamini-Hochberg procedure with a False Discovery Rate of 0.05 given the exploratory and hypothesis-generating nature of this study. Only the corrected P-values smaller than 0.05 (i.e. regarded significant) are reported in Table 2, Table 3, Table 4. Statistical analyses were performed using IBM SPSS Statistics version 23.0 and Microsoft Office Excel 2010.

**Table 2:**
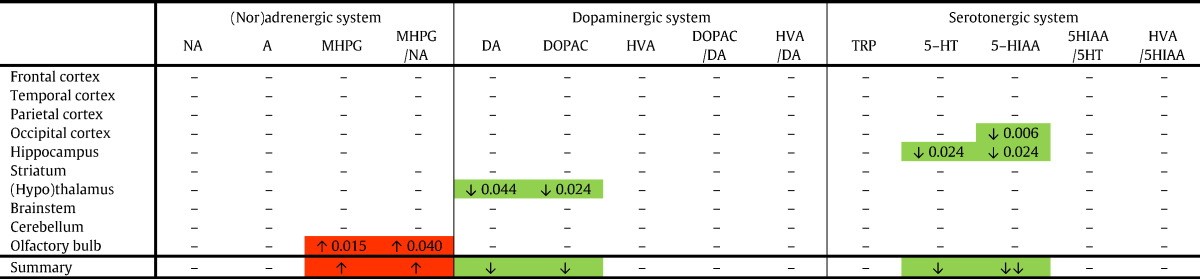
Young Ts65Dn vs. young WT_Ts65Dn_ mice (aneuploidy effect).

Red boxes indicate a significant increase, and green boxes depict a significant decrease for a compound or ratio in young Ts65Dn vs. young WT_Ts65Dn_ mice. The summary row indicates the consequent overall increase (red) or decrease (green). 5-HIAA, 5-hydroxyindoleacetic acid; 5-HT, serotonin; A, adrenaline; DA, dopamine; DOPAC, 3-4-dihydroxyphenylacetic acid; HVA, homovanillic acid; MHPG, 3-methoxy-4-hydroxyphenylglycol; NA, noradrenaline.

**Table 3:**
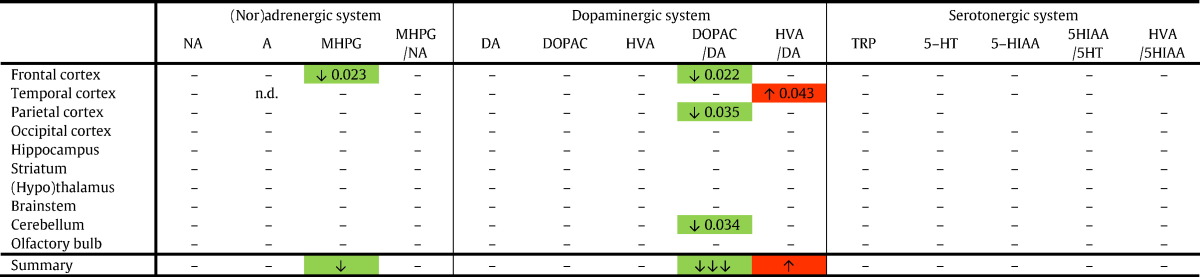
Aged Ts65Dn vs. aged WT_Ts65Dn_ mice (aneuploidy effect).

Red boxes indicate a significant increase, and green boxes depict a significant decrease for a compound or ratio in aged Ts65Dn vs. aged WT_Ts65Dn_ mice. The summary row indicates the consequent overall increase (red) or decrease (green). 5-HIAA, 5-hydroxyindoleacetic acid; 5-HT, serotonin; A, adrenaline; DA, dopamine; DOPAC, 3-4-dihydroxyphenylacetic acid; HVA, homovanillic acid; MHPG, 3-methoxy-4-hydroxyphenylglycol; NA, noradrenaline; n.d., not detectable.

**Table 4:**
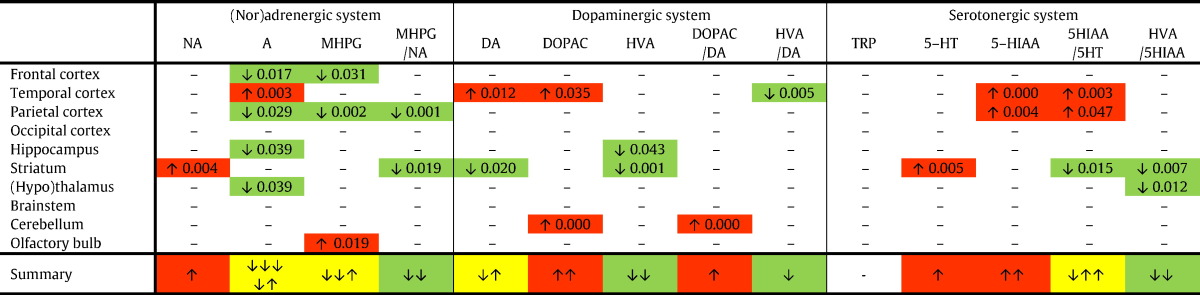
Aged Ts65Dn vs. young Ts65Dn mice (aging effect).

Red boxes indicate a significant increase, and green boxes depict a significant decrease for a compound or ratio in aged Ts65Dn vs. young Ts65Dn mice. The summary row indicates the consequent overall increase (red) or decrease (green). The yellow boxes indicate that the direction of the change differed between the different regions: the arrows illustrate whether the direction of change is ex aequo (e.g. ↓↑) or inclines towards an increase (e.g. ↓↑↑) or decrease (e.g. ↓↓↑). 5-HIAA, 5-hydroxyindoleacetic acid; 5-HT, serotonin; A, adrenaline; DA, dopamine; DOPAC, 3-4-dihydroxyphenylacetic acid; HVA, homovanillic acid; MHPG, 3-methoxy-4-hydroxyphenylglycol; NA, noradrenaline.

## 3 Results

Each monoaminergic compound was detected and quantified in all ten brain regions. Based on these concentrations, five accompanying ratios were calculated: MHPG/NA (reflecting noradrenergic metabolism), DOPAC/DA and HVA/DA (reflecting dopaminergic catabolism), 5-HIAA/5-HT (reflecting serotonergic catabolism) and, finally, HVA/5-HIAA (indicating the effect of serotonergic inhibition on dopaminergic neurotransmission). In the supplementary material (S1-S6), the concentrations and ratios for each brain region are provided per experimental group (median and quartiles, including the number of samples in which a specific compound was detected). All compounds and ratios were compared between young Ts65Dn vs. young WT_Ts65Dn_ (Table 2), aged Ts65Dn vs. aged WT_Ts65Dn_ (Table 3), aged Ts65Dn vs. young Ts65Dn (Table 4), aged WT_Ts65Dn_ vs. young WT_Ts65Dn_ (Table 5), and young Dp1Tyb vs. young WT_Dp1Tyb_ mice. Since the Ts65Dn and Dp1Tyb mouse models have different genetic backgrounds and were bred and housed in two different institutes, these DS models are not compared with each other, but only to their age-matched WT littermates (same background, same conditions). Significant P-values of the individual group comparisons are provided in Tables 2–4.

**Table 5:**
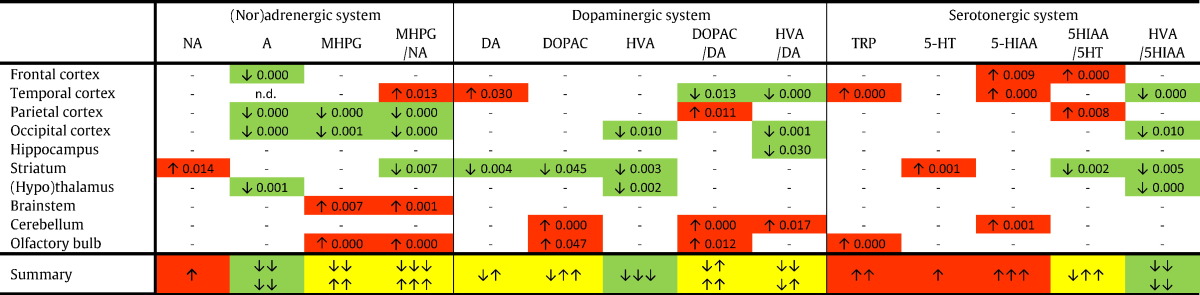
Aged WT_Ts65Dn_ vs. young WT_Ts65Dn_ mice (aging effect).

Red boxes indicate a significant increase, and green boxes depict a significant decrease for a compound or ratio in aged WT_Ts65Dn_ vs. young WT_Ts65Dn_ mice. The summary row indicates the consequent overall increase (red) or decrease (green). The yellow boxes indicate that the direction of the change differed between the different regions: the arrows illustrate whether the direction of change is ex aequo (e.g. ↓↑) or inclines towards an increase (e.g. ↓↑↑) or decrease (e.g. ↓↓↑). 5-HIAA, 5-hydroxyindoleacetic acid; 5-HT, serotonin; A, adrenaline; DA, dopamine; DOPAC, 3-4-dihydroxyphenylacetic acid; HVA, homovanillic acid; MHPG, 3-methoxy-4-hydroxyphenylglycol; NA, noradrenaline; n.d., not detectable.

### 3.1 Effect of aneuploidy

#### 3.1.1 Ts65Dn vs. WT_Ts65Dn_ mice

To determine the effect of the aneuploidy on the monoamine neurotransmitters and their metabolites, we compared Ts65Dn mice with their WT_Ts65Dn_ littermates. First of all, we compared the young animals (Table 2): MHPG and the accompanying MHPG/NA ratio in the olfactory bulb were significantly higher in young Ts65Dn than in young WT_Ts65Dn_ mice. No other significant changes in the (nor)adrenergic system were found. Concerning the dopaminergic system, DA and DOPAC were significantly lower in the (hypo)thalamus of Ts65Dn compared to WT_Ts65Dn_ mice, but not in other regions. With respect to the serotonergic compounds, decreased levels were observed for 5-HIAA and 5-HT in the hippocampus, and 5-HIAA in the occipital cortex of young Ts65Dn compared to young WT_Ts65Dn_ mice.

Secondly, we compared the aged Ts65Dn mice with their aged WT_Ts65Dn_ littermates (Table 3). Apart from a significant reduction of MHPG in the frontal cortex of Ts65Dn mice, no other significant changes were observed for the (nor)adrenergic system in any of the brain regions. Although DA, DOPAC and HVA did not differ significantly between the groups, the subsequent ratios turned out to be significantly altered: the DOPAC/DA ratio was lower in the frontal cortex, parietal cortex, and cerebellum of Ts65Dn mice (indicating reduced DA catabolism), while the ratio HVA/DA in the temporal cortex was increased (indicating increased DA metabolization) in aged Ts65Dn compared to aged WT_Ts65Dn_ mice. No differences were found in the serotonergic system.

#### 3.1.2 Young Dp1Tyb vs. young WT_Dp1Tyb_ mice

In addition to the Ts65Dn mouse model, we also assessed the monoaminergic profile of young Dp1Tyb mice in comparison with their WT_Dp1Tyb_ littermates: only the NA concentration in the occipital cortex was significantly increased in Dp1Tyb mice compared to WT_Dp1Tyb_ (P = 0.047), while none of the other monoamines, metabolites or ratios were significantly altered between both groups.

### 3.2 Effect of aging

#### 3.2.1 Aged Ts65Dn vs. young Ts65Dn mice

To study the effect of aging in Ts65Dn mice, we compared two age groups: Table 4 depicts the significant differences between aged Ts65Dn (~ 12–13 months) and their younger counterparts (~ 3–5.5 months). To start with the noradrenergic system, NA concentrations only differed in the striatum, but not in other regions. Adrenaline, in contrast, was substantially affected by aging: significantly lower levels were found in the frontal cortex, parietal cortex, hippocampus and (hypo)thalamus, and higher levels in the temporal cortex. Similarly, MHPG was reduced in the frontal and parietal cortex, but was elevated in the olfactory bulb. Consequently, the ratio MHPG/NA in the parietal cortex and the striatum was decreased in aged compared to young Ts65Dn mice. Overall, adrenaline, MHPG and the MHPG/NA ratio were predominantly reduced with aging.

In contrast to these results, the changes in the dopaminergic system are less consistent: DA was increased in the temporal cortex, but decreased in the striatum of aged vs. young Ts65Dn mice. DOPAC was significantly higher in the temporal cortex and cerebellum, while lower HVA concentrations were found in the hippocampus and striatum. The resulting DOPAC/DA and HVA/DA ratios were not altered for the different brain regions, apart from an increased DOPAC/DA ratio in the cerebellum and a decreased HVA/DA ratio in the temporal cortex.

Concerning the serotonergic system, 5-HIAA and the 5-HIAA/5-HT ratio were increased in the temporal and parietal cortex, pointing at increased serotonergic turnover. In the striatum, 5-HT was increased and the ratio 5-HIAA/5HT reduced, indicating reduced serotonergic catabolism. Finally, the inhibitory effect of the serotonergic system on the dopaminergic system is reflected by the HVA/5-HIAA ratio, which turned out to be significantly lower in the striatum and the (hypo)thalamus of aged Ts65Dn mice compared to their younger counterparts.

#### 3.2.2 Aged WT_Ts65Dn_ vs. young WT_Ts65Dn_ mice

To evaluate whether the observed changes in aged Ts65Dn vs. young Ts65Dn mice are specific for this model, i.e. relate to the triplicated genes or to aging in general, we also compared aged WT_Ts65Dn_ mice (~ 12–13 months) vs. young (~ 3–5.5 months) animals (Table 5). In the (nor)adrenergic system, striatal NA concentrations were higher in aged than in young WT_Ts65Dn_ mice, resembling the observed increase in the striatum of Ts65Dn mice. Furthermore, adrenaline was lower in the frontal cortex, parietal cortex, occipital cortex and (hypo)thalamus of aged WT_Ts65Dn_ mice than in their younger counterparts, which again (largely) resembles the aforementioned findings in the Ts65Dn model. In the aged animals, MHPG levels were significantly reduced in cortical regions (parietal and occipital cortex), but increased in brainstem and olfactory bulb. Accordingly, the ratio MHPG/NA decreased in parietal and occipital cortex, as well as in the striatum, and increased in the temporal cortex, brainstem and olfactory bulb.

In accordance with the Ts65Dn results, DA levels were significantly higher in the temporal cortex and lower in the striatum of aged vs. young WT_Ts65Dn_ mice. DOPAC and HVA were both decreased in the striatum as well. In addition, aged WT_Ts65Dn_ animals showed increased DOPAC levels in the cerebellum and olfactory bulb, and reduced HVA concentrations in the occipital cortex and (hypo)thalamus, compared to their younger counterparts. Concerning the ratios, DOPAC/DA was predominantly increased (parietal cortex, cerebellum and olfactory bulb, but decreased in temporal cortex), while the ratio HVA/DA was mainly decreased (temporal cortex, occipital cortex and hippocampus, but increased in cerebellum).

Finally, an overall increase of serotonergic compounds became apparent when comparing aged vs. young WT_Ts65Dn_ mice. Significant increases were found for tryptophan in the temporal cortex and olfactory bulb, 5-HT in the striatum, 5-HIAA in the frontal cortex, temporal cortex and cerebellum and the ratio 5-HIAA/5-HT in the frontal cortex and parietal cortex. In contrast, the ratio 5-HIAA/5HT was decreased in the striatum. As in Ts65Dn mice, the ratio HVA/5-HIAA was generally reduced in aged WT_Ts65Dn_ mice, indicating enhanced serotonergic inhibition on the dopaminergic system.

## 4 Discussion

Using a previously optimized and validated RP-HPLC methodology (Van Dam et al., 2014), we detected noradrenergic (NA, adrenaline and MHPG), dopaminergic (DA, DOPAC and HVA) and serotonergic (TRP, 5-HT and 5-HIAA) compounds in ten regionally dissected brain regions to establish the monoaminergic profiles of Dp1Tyb and Ts65Dn mice and their WT littermates. First of all, we studied the effect of aneuploidy in both models. Comparing young Dp1Tyb with young WT_Dp1Tyb_ showed that only occipital NA levels differed significantly, suggesting that the additional genes in Dp1Tyb mice hardly affected monoamine concentrations at this age. Similarly, Table 2, Table 3 depict the comparisons between Ts65Dn mice and WT_Ts65Dn_ littermates, and do not show generalized monoaminergic dysregulation either, even though a few more changes were regarded significant than in the Dp1Tyb mouse model. Accordingly, the genetic overload in these DS mouse models barely affected the absolute monoaminergic concentrations at the analyzed ages. Secondly, we studied the effect of aging within Ts65Dn and WT_Ts65Dn_ mice comparing aged (~ 12–13 months) vs. young animals (~ 3–5.5 months). The overall patterns of monoaminergic alterations appeared to be relatively similar in both genotypes, though more observed changes (especially in the ratios) were regarded significant for WT_Ts65Dn_ mice. Indeed, these overall patterns, rather than the individual concentration changes in a specific region, are most relevant in this exploratory study. In the next section, the main patterns and changes will be highlighted and discussed.

### 4.1 Noradrenergic system

The LC, located in the pons near the fourth ventricle, is the major source of NA in the brain. The ascending noradrenergic neurons originating from this small nucleus project to an extensive number of (sub)cortical brain regions, including the frontal cortex, hippocampus, striatum, (hypo)thalamus, cerebellum and olfactory bulb, mediating, among others, arousal, attention and contextual memory. Importantly, LC neurons are the sole source of NA for the cortex, hippocampus, cerebellum and most of the thalamus (Aston-Jones and Cohen, 2005, Trillo et al., 2013, Vermeiren et al., 2016). A loss of LC neurons may thus especially affect these brain areas.

Previously, such a loss was reported in Ts65Dn mice of 6, 12 and 18 months but not in younger Ts65Dn mice (aged 3 and 4 months) compared to age-matched WT_Ts65Dn_ mice (Fortress et al., 2015, Lockrow et al., 2011, Salehi et al., 2009). Here, we did not observe significant NA concentration changes between Ts65Dn and WT_Ts65Dn_ in both age groups for the brainstem (including LC) or any of the noradrenergic projection regions (Table 2, Table 3). Indeed, immunohistochemical characterization of the catecholaminergic nuclei (anti-tyrosine hydroxylase antibodies) previously showed intense staining of the LC in both Ts65Dn and control mice (3 months) without evident differences between both groups, indicating that the additional genes in Ts65Dn did not influence the early LC development (Megías et al., 1997). This is further supported by human findings: NA levels were not significantly altered in the frontal cortex of fetal DS tissue (20 weeks) as compared to age-matched non-DS fetuses (Whittle et al., 2007). Similarly, the genetic overload in Dp1Tyb mice does not appear to affect NA levels other than an increase in the occipital cortex in comparison with WT_Dp1Tyb_ mice – that is the only significant difference in all compounds and regions in this strain. Next, comparing trisomic mice with their WT littermates revealed no significant changes in adrenaline, whereas MHPG was only altered in the olfactory bulb of young Ts65Dn vs. WT_Ts65Dn_ and frontal cortex of aged Ts65Dn vs. WT_Ts65Dn_ mice. The latter resembles the reduced levels of MHPG, which freely diffuses over the blood-brain barrier, in serum of elderly DS individuals as compared to non-DS controls (Dekker et al., 2015a), and may relate to the previously reported deficits in (pre)frontal cortex-mediated spatial working memory in Ts65Dn mice aged 9–12 months (Dudchenko, 2004, Faizi et al., 2011, Yoon et al., 2008). Nevertheless, it appears that the aneuploidy had a very limited effect on the (nor)adrenergic system.

In contrast, the effect of aging (i.e. comparing aged vs. young animals of the same genotype) is pronounced for adrenaline and MHPG, but not for NA. We did not find age-related NA alterations other than the increased NA levels in the striatum (Table 4, Table 5). Interestingly, the precursor of NA, that is DA, was decreased in the striatum of the aged mice, possibly pointing at an increased turnover of DA into NA (Table 4, Table 5). The virtually unaltered NA levels between the young and aged animals possibly relate to enhanced NA synthesis in the remaining noradrenergic cells, i.e. a compensatory mechanism (Schapiro et al., 1987). With progressive neuronal loss in the LC during aging (Fortress et al., 2015, Lockrow et al., 2011, Salehi et al., 2009), which is also observed in DS (Mann et al., 1987b, Marcyniuk et al., 1988), the increasingly smaller number of remaining cells will not be able to keep noradrenergic neurotransmission at level, causing NA concentrations to start decreasing from a certain moment onwards. Indeed, Salehi et al. found an age-related decrease in hippocampal NA concentrations, which was only significant for Ts65Dn vs. WT_Ts65Dn_ at 18 months. Interestingly, immunostaining for the β1-adrenergic receptor, present on postsynaptic targets of LC neurons in the hippocampus, revealed an increased size (at 3 and 6 months) and number (at 6 months) of immune-reactive cells in Ts65Dn mice compared with age-matched WT_Ts65Dn_ mice, pointing at a postsynaptic compensation for the age-related decrease in NA levels (Salehi et al., 2009). This may resemble the human situation, where significantly reduced NA levels have been reported later in life in several (sub)cortical post-mortem brain samples of elderly DS individuals presenting AD neuropathology as compared to controls (Godridge et al., 1987, Reynolds and Godridge, 1985, Risser et al., 1997, Yates et al., 1981).

Conversely, adrenaline levels appear to be substantially altered over time in both Ts65Dn and WT_Ts65Dn_ mice. A relatively similar pattern was observed for both genotypes: older animals had significantly decreased adrenaline levels in cortical regions and (hypo)thalamus (Table 4, Table 5). Hippocampal adrenaline was also reduced in aged Ts65Dn. Adrenaline is formed by methylation of NA by the rate-limiting enzyme phenylethanolamine *N*-methyltransferase (PNMT, Fig.1). The reduced adrenaline levels are not likely due to reduced NA levels, given the fact that NA levels were unaltered. Instead, lower adrenaline levels may be due to a decrease in the amount or activity of PNMT enzyme. Burke and co-workers, for instance, described a strong reduction of enzymatic activity due to lower amounts of PNMT enzymes in human AD brain tissue as compared to controls (Burke et al., 1987). Since the current study methodology did not enable enzymatic measurements (the small tissue samples were entirely used for RP-HPLC analyses), future studies need to elucidate whether PNMT plays a role in the adrenergic changes in these mice.

MHPG levels were also affected by aging: reduced adrenaline levels in the frontal and parietal cortex of aged Ts65Dn mice, and parietal and occipital cortex of aged WT_Ts65Dn_ mice likely resulted in the decreased MHPG levels in these regions. Whereas MHPG and the MHPG/NA ratio generally decreased with aging in Ts65Dn, the pattern for WT_Ts65Dn_ mice was more diffuse. NA and adrenaline are converted into MHPG through the enzymes monoamine oxidase (MAO, Fig.1) and catechol-*O*-methyltransferase (COMT, Fig.1). Previously, MAO activity in platelets of DS individuals was found to be reduced (Benson and Southgate, 1970, Fowler et al., 1981) or unaltered (Lott et al., 1972), whereas COMT activity was higher (Gustavson et al., 1973) or unaltered (Brahe et al., 1985) in DS erythrocytes. The COMT enzyme is a methyl transferase, using the methyl group of S-adenosylmethionine (SAM). The levels of the methyl donor SAM are reduced in DS (Dekker et al., 2014), suggesting that COMT activity might be reduced in DS as well. Nevertheless, it remains to be elucidated whether the amount or activity of MAO and COMT change over time in these mice.

Taken together, the (nor)adrenergic system was not substantially affected by the aneuploidy in Ts65Dn or Dp1Tyb mice. However, aging revealed strong alterations in multiple brain regions. Since LC degeneration and altered (nor)adrenergic neurotransmission are implicated in DS and AD, others have studied the effect of (nor)adrenergic agonists in (aged) Ts65Dn mice as potential therapeutic strategies (Phillips et al., 2016). Faizi and colleagues, for instance, reported reproducible learning and memory deficits in Ts65Dn mice of 9–12 months of age that could be restored by administering the β1-adrenergic receptor partial agonist xamoterol, thus suggesting that such cognitive deficits are (in part) mediated by the (nor)adrenergic system (Faizi et al., 2011). Similarly, xamoterol and the NA-prodrug l-DOPS reversed contextual learning impairment in 6-month-old Ts65Dn mice (Salehi et al., 2009). Moreover, the long-acting β2-adrenergic receptor agonist formoterol significantly improved cognitive capacities, synaptic density and complexity of the dendritic tree of new dentate granule cells in the hippocampus of Ts65Dn mice (5–6 months) (Dang et al., 2014). More recently, Fortress and colleagues demonstrated LC degeneration in 12-month-old Ts65Dn mice, and found that selective stimulation of the remaining noradrenergic LC neurons using designer receptors exclusively activated by designer drugs (DREADDs) restored memory function (Fortress et al., 2015).

In the context of the high risk for AD in DS, it is important to note that the amyloid precursor protein (APP) gene located on HSA21 is also triplicated in Ts65Dn mice, but that the APP cleavage product amyloid-beta (Aβ) does not accumulate into plaques in Ts65Dn mice like in the human condition (Gardiner, 2015, Reeves et al., 1995). Consequently, Ts65Dn is primarily a model of the developmental trisomy 21-related deficits in DS and can be used to study the effect of *APP* overexpression, but not Aβ accumulation, with aging. Interestingly, Salehi and colleagues found that the loss of LC neurons was eliminated by deleting the third copy of the *APP* gene in Ts65Dn mice, thus indicating that triplicated *APP* plays an essential role in LC degeneration (Salehi et al., 2009). Indeed, a comparable cell loss of approximately 60% was found in post-mortem LC samples from elderly DS (57–59 years) and AD patients (61–82 years) in the general population compared to healthy controls (60–76 years) (German et al., 1992). Importantly, in both AD and older DS samples, neuronal loss mainly affected the neurons projecting to the cortex, while the non-cortical projecting cells were spared (German et al., 1992, Marcyniuk et al., 1988). Similarly, LC neuronal loss in Ts65Dn mice at 12 months was mainly restricted to the rostral neurons projecting to the forebrain and hippocampus, while the caudal regions were spared (Lockrow et al., 2011). That might explain the discrepancy between our finding of decreased cortical MHPG levels and the increased MHPG concentrations in the brainstem with aging. Cautious interpretation of the results is warranted in relation to findings in aged post-mortem DS samples: virtually all DS individuals have omnipresent AD-pathology (i.e. Aβ plaques and tau tangles) in their brain from the age of 40 years onwards (Mann, 1988), complicating the comparison between findings in DS and Ts65Dn mice that do not model human AD neuropathology in DS.

Interestingly, LC degeneration does not only affect noradrenergic neurotransmission, but also impacts cholinergic signaling (Lockrow et al., 2011). Basal forebrain cholinergic neurons innervate the amygdala, hippocampus and neocortex and are strongly implicated in attention and memory processes. A significant loss of cholinergic cells has been found in AD and DS (Casanova et al., 1985, Mann et al., 1985, Mufson et al., 2003) and in Ts65Dn mice by 12 months of age (Cooper et al., 2001). LC degeneration in Ts65Dn mice appears to precede the onset of cholinergic degeneration (Phillips et al., 2016). Indeed, Lockrow and colleagues demonstrated that neurotoxin-induced NA depletion resulted in accelerated cholinergic degeneration in Ts65Dn mice, but not in WT_Ts65Dn_ mice. Learning and memory deficits were aggravated in Ts65Dn mice as well (Lockrow et al., 2011). In the current study, we report an overall decrease of adrenaline, MHPG and the MHPG/NA ratio with aging in Ts65Dn mice, which may possibly contribute to cholinergic pathology in this mouse model. Future studies should consider studying monoaminergic alterations in relation to cholinergic deficits.

In conclusion, the (nor)adrenergic system was mainly affected by aging rather than by aneuploidy. Comparing aged vs. young animals revealed altered patterns across multiple brain regions. In particular, adrenaline and MHPG differed in most cortical regions as well as in a few subcortical structures. In Ts65Dn an overall decrease of adrenaline, MHPG and the MHPG/NA ratio became apparent with aging. In WT_Ts65Dn_ mice, adrenaline decreased with aging as well, while the directions of change for MHPG and MHPG/NA were less straightforward.

### 4.2 Dopaminergic system

DA, derived from l-tyrosine (Fig. 1), is mainly produced by the substantia nigra (SN) and ventral tegmental area (VTA) located in the mesencephalon. The SN primarily projects to the striatum (hence referred to as the nigrostriatal pathway), whereas the VTA projects to the limbic (mesolimbic pathway) and cortical areas (mesocortical pathway). DA is involved in regulating motor activity, emotions, reward and has been associated with cognition (Lanari et al., 2006, Nieoullon, 2002, Trillo et al., 2013).

Studying the effect of aneuploidy on the dopaminergic system showed that the levels of DA, DOPAC and HVA are virtually unaltered, apart from decreased DA and DOPAC levels in the (hypo)thalamus of young Ts65Dn vs. WT_Ts65Dn_ mice. Indeed, immunohistochemical staining of the dopaminergic neurons (anti-tyrosine hydroxylase antibodies) in the SN and VTA of 3-month-old Ts65Dn mice and WT_Ts65Dn_ mice revealed similar reaction intensities in both genotypes. The unaltered morphology and number of reactive cells between Ts65Dn and WT_Ts65Dn_ led the authors to conclude that the triplicated genes in Ts65Dn do not affect the development of dopaminergic nuclei (Megías et al., 1997). In young Ts65Dn mice, we found reduced DA levels in the (hypo)thalamus. In the human situation, levels of DA and the DA receptor 1-containing complex were significantly lower in the frontal cortex of DS fetuses than in age-matched control fetuses (Keihan Falsafi et al., 2016, Whittle et al., 2007). Furthermore, the lack of significant alterations between the older Ts65Dn and WT_Ts65Dn_ mice resembles the lack of significant changes in multiple post-mortem brain samples of DS individuals vs. controls (Godridge et al., 1987).

In contrast to the relatively consistent age-related patterns of change in the (nor)adrenergic (section 4.1) and serotonergic systems (section 4.3), the results for the dopaminergic system are less consistent over the months for the different compounds/regions. In the human situation, Mann and colleagues reported neuronal loss in the VTA, but not in the SN of aged DS (51–65 years) and AD patients (53–89 years) (Mann et al., 1987a). Indeed, comparison of older and younger DS samples revealed no significant cell loss or reduced nucleolar volume in the SN over time (Mann et al., 1987b). In Ts65Dn mice of 3 months, neuronal loss was absent (Megías et al., 1997), but whether this is true for aged Ts65Dn mice remains unclear. With aging, this study showed that DA increased in the temporal cortex, but decreased in the striatum for aged vs. young Ts65Dn mice, as well as for aged vs. young WT_Ts65Dn_ mice. In mice, striatal neurons receive input from both the SN and VTA (Zeiss, 2005). The finding that DOPAC is increased in various regions, while HVA is decreased may relate to changes in MAO or COMT activity (Fig. 1). COMT transfers a methyl group of SAM to DOPAC (3-*O*-methylation) resulting in HVA (Meiser et al., 2013). As described before, the levels of the methyl donor SAM are reduced in DS (Dekker et al., 2014), possibly affecting DOPAC methylation and thus HVA levels.

In short, the dopaminergic system was barely affected by the triplicated HSA21-homologous genes in Ts65Dn or Dp1Tyb mice compared to their WT littermates. Aging resulted in altered DA, DOPAC and HVA levels, but further studies are required to mechanistically demonstrate the cause of these alterations.

### 4.3 Serotonergic system

The indolamine 5-HT, derived from l-tryptophan (Fig. 1), is produced in various cell groups in the medulla, pons and mesencephalon. The majority of ascending serotonergic neurons arise from the dorsal and median raphe nuclei. In particular, the dorsal raphe nuclei project to the cortex and striatum, while the median raphe nuclei target the cortex, hippocampus and hypothalamus (Trillo et al., 2013). The main role of the serotonergic system is (behavioral) inhibition, and regulation of mood and aggression, for instance (Lanctôt et al., 2001).

Here, we did not find widespread serotonergic changes between Ts65Dn and WT_Ts65Dn_ mice. This is in accordance with the conclusion of Megías et al. that the development of the serotonergic system is not evidently influenced by the triplicated genes in Ts65Dn mice. Indeed, their immunohistochemical results demonstrated a similar distribution of serotonin-immunoreactive cells (anti-serotonin antibodies) in the medial and dorsal raphe nuclei of Ts65Dn and WT_Ts65Dn_ mice at 3 months of age (Megías et al., 1997). 5-HT has been implicated in the impaired hippocampal neurogenesis and dendritic hypotrophy in Ts65Dn mice. Ts65Dn mice, similar to DS individuals, have a smaller hippocampus indeed. Prenatal and neonatal treatment with the selective serotonin reuptake inhibitor fluoxetine was found to normalize hippocampal neurogenesis and restore dendritic architecture, hippocampal cellularity and memory functions, even 1.5–3 months after treatment cessation (reviewed in: Stagni et al., 2015). Although we do not see many serotonergic changes between both genotypes, we did find significantly reduced 5-HT and 5-HIAA levels in the hippocampus of young Ts65Dn compared to young WT_Ts65Dn_ mice.

Whether these findings reflect the human situation remains unclear due to a lack of (post-mortem) studies in young DS individuals. Reduced 5-HT and 5-HIAA levels have, indeed, been reported in post-mortem DS hippocampus samples (Godridge et al., 1987, Reynolds and Godridge, 1985). Other studies confirmed the pattern of lower 5-HT and 5-HIAA concentrations in DS (Risser et al., 1997, Seidl et al., 1999, Whittle et al., 2007, Yates et al., 1986). These human post-mortem studies were mainly performed on samples of elderly DS individuals presenting AD neuropathology. For instance, Yates et al. studied six DS brains over 50 years of age with numerous plaques and tangles in the cortex and reported significantly decreased 5-HT levels in amygdala, caudate nucleus (part of striatum) and cingulate cortex, but no such change was found in a 27-year-old DS case without AD pathology (Yates et al., 1986). Considering fetal samples, however, Whittle et al. found reduced 5-HT and 5-HIAA in the frontal cortex of fetal DS tissue as compared to age-matched control aborted fetuses (Whittle et al., 2007), suggesting that this reduction is not related to the AD pathology, but to the trisomy 21 itself. To which extent the murine findings thus resemble the human situation is questionable given the fact that Aβ does not accumulate into plaques in these mice, and comparison of aged Ts65Dn with aged WT_Ts65Dn_ did not yield significant alterations in any serotonergic compound or region.

Studying the effect of aging within each genotype revealed a more pronounced pattern: an overall increase of serotonergic compounds with aging. This is in remarkable contrast with the aforementioned reduced serotonergic levels in the elderly DS brain samples. Indeed, comparison of older DS brains (with AD pathology) with young DS brains (without/minimal AD pathology) revealed a significant decrease in the number of cells and the nucleolar volume with aging (Mann et al., 1987b). Neuronal loss has been reported in the dorsal tegmental nucleus of the raphe nuclei in six DS patients over 50 years, as well as in AD patients in the general population (Mann et al., 1985). The activity of the tryptophan hydroxylase enzyme (Fig. 1), key to 5-HT biosynthesis, is reduced in AD as well (Trillo et al., 2013), but whether this is also true for DS and Ts65Dn is unclear. The link between AD pathology and 5-HT levels is further supported by the finding that the increased levels of APP-derived βCTF peptide could be normalized in adult Ts65Dn mice through fluoxetine treatment (Stagni et al., 2015). Therefore, the underlying mechanism of serotonergic increase with aging in these mice should be studied more extensively.

### 4.4 Future implications

Cautious interpretation of the results is required, because the underlying causes for (the lack of) monoaminergic alterations remain to be elucidated. The virtually unaltered NA levels and the likely presence of a compensatory mechanism, for instance, illustrate the necessity to study monoaminergic alterations over a longer period of time. Therefore, we propose that future studies should evaluate mice in a larger number of age groups, for instance at 3, 6, 9, 12, 15, 18 and 21 months. Immunohistochemical staining of (nor)adrenergic, dopaminergic and serotonergic neurons, and measurements of the amount and activity of key enzymes like PNMT, MAO and COMT (Fig. 1) in mice of different ages would be a valuable addition to understand the age-related changes. Despite the fact that the age ranges of the young Ts65Dn and Dp1Tyb mice in the current study slightly overlapped, the young Ts65Dn mice were, on average, a few weeks older than the Dp1Tyb mice. Although it was not the aim to compare both models with each other (different genetic backgrounds and different institutes), it could be considered a limitation of this study. Comparative age-matched studies should certainly be considered in the future, providing that the mice are bred and housed at the same location. Moreover, the novel Dp1Tyb mouse model has not been fully characterized yet. Dp1Tyb mice present congenital heart defects comparable to DS (Lana-Elola et al., 2016). Brain morphology and behavioral deficits have not been reported so far, although brain development and cognitive deficits in Dp1Tyb mice are expected to resemble those reported in the essentially similar Dp(16)1Yey mouse model (Goodliffe et al., 2016, Yu et al., 2010). Future studies should specifically evaluate the behavioral phenotype (DS-related deficits) of Dp1Tyb mice in young, adult and aged life.

Currently, it is complex to evaluate the monoaminergic construct validity of the mouse models in comparison with the human situation, because of the lack of comprehensive post-mortem brain studies in DS. The few available monoaminergic studies were conducted twenty to forty years ago and have various methodological limitations, including (1) long post-mortem delays (PMD), (2) small sample sizes, and (3) absence of DS brains free of extensive AD pathology. First, long PMDs over 30 h, for instance, were not unusual (Godridge et al., 1987, Risser et al., 1997). Indeed, PMD is a confounding factor since it may affect the (regional) concentrations of specific monoamines and metabolites (Kontur et al., 1994, Van Dam et al., 2014). Therefore, we have submersed the mice in liquid nitrogen within minutes to stop further monoaminergic turnover. Secondly, (very) small sample sizes were used, ranging from 2 to 8 individuals (Godridge et al., 1987, Reynolds and Godridge, 1985, Risser et al., 1997, Yates et al., 1981), thus complicating the generalizability of the human results. Thirdly, mainly older DS brains with virtually omnipresent AD-like neuropathology were studied (Mann, 1988), making it rather complex to disentangle the effects of AD pathology, normal aging, and trisomy 21. To establish whether the monoaminergic phenotypes of DS mouse models reflect alterations in the human situation, we should study post-mortem human brain samples in greater detail, and thus prioritize the collection of these samples through, for instance, multicenter brain bank initiatives. In short, monoamine neurotransmitter systems have not been comprehensively studied in the human DS situation, illustrating the need to be careful with drawing conclusions at this stage about the monoaminergic construct validity of Ts65Dn and Dp1Tyb mouse models.

With respect to the high risk for AD in DS, the scientific community is in great need of animal models that integrate DS and AD phenotypes, i.e. a model for the neurodevelopmental deficits in DS (such as Ts65Dn) that also develops AD pathology and related neurodegeneration over time. Clearly, overexpression of wild-type *APP* is not sufficient for the development of vast AD neuropathology in these mice (Wiseman et al., 2015). Indeed, we studied the effect of triplicated genes, including *APP*, as well as the effect of normal aging in these mice that model DS, but not AD. Novel rodent models for AD in DS are being developed, but these are not available yet. Such models would be of great value to study monoaminergic changes due to ‘pathologic’ aging.

## 5 Conclusion

To the best of our knowledge, this study is the first to comprehensively quantify monoamine neurotransmitters and metabolites in ten regionally dissected brain regions in two DS mouse models in comparison with their WT littermates. Monoaminergic concentrations were barely altered by the aneuploidy in young adult Ts65Dn and Dp1Tyb mice compared with their WT littermates. Aging to 12–13 months, however, revealed strong alterations in the monoaminergic compounds in multiple brain regions in both Ts65Dn and WT mice. Future studies should focus on replicating these age-related monoaminergic alterations and elucidate the underlying causes. Post-mortem studies with human DS brain samples are necessary to validate the monoaminergic construct validity of the Ts65Dn and Dp1Typ mouse models of DS.

## Appendix A Supplementary data

Supplementary tables Image 1
