# Supplementary material for: Aging rather than aneuploidy affects monoamine neurotransmitters in brain regions of Down syndrome mouse models
Source: Neurobiol Dis. 2017 Sep;105:235–44. doi: 10.1016/j.nbd.2017.06.007 (PMC5536154; doi:10.1016/j.nbd.2017.06.007)
Supplement: Supplementary file 1 — Supplementary tables [file mmc1.docx]

**Table S1: Concentrations of monoaminergic compounds and ratios in young Ts65Dn mice**

|  |  | **(nor)adrenergic system** | | | | **dopaminergic system** | | | | | **serotonergic system** | | | | |
| --- | --- | --- | --- | --- | --- | --- | --- | --- | --- | --- | --- | --- | --- | --- | --- |
|  |  | **NA**  **(ng/ml)** | **A**  **(ng/ml)** | **MHPG**  **(ng/ml)** | **MHPG**  **/NA** | **DA**  **(ng/ml)** | **DOPAC**  **(ng/ml)** | **HVA**  **(ng/ml)** | **DOPAC**  **/DA** | **HVA**  **/DA** | **TRP**  **(ng/ml)** | **5-HT**  **(ng/ml)** | **5-HIAA**  **(ng/ml)** | **5HIAA**  **/5HT** | **HVA**  **/5HIAA** |
| Frontal cortex | 25% | 377.9 | 14.8 | 67.7 | 0.16 | 28.8 | 19.1 | 48.5 | 0.34 | 0.93 | 3056.4 | 513.8 | 111.7 | 0.20 | 0.36 |
|  | 50% | 439.5 | 20.3 | 85.0 | 0.18 | 42.6 | 22.5 | 55.9 | 0.56 | 1.37 | 3321.0 | 546.7 | 138.6 | 0.22 | 0.45 |
|  | 75% | 532.4 | 26.3 | 98.2 | 0.21 | 73.7 | 30.8 | 78.8 | 0.75 | 1.82 | 3671.8 | 650.4 | 154.3 | 0.28 | 0.54 |
|  | *N* | *21* | *21* | *21* | *21* | *21* | *21* | *21* | *21* | *21* | *21* | *21* | *21* | *21* | *21* |
| Temporal cortex | 25% | 389.4 | 3.4 | 65.8 | 0.14 | 36.9 | 31.4 | 76.3 | 0.58 | 1.43 | 3091.6 | 376.7 | 128.4 | 0.31 | 0.55 |
|  | 50% | 446.5 | 5.4 | 77.6 | 0.18 | 55.5 | 51.6 | 108.9 | 0.83 | 1.71 | 3374.6 | 436.2 | 157.6 | 0.34 | 0.67 |
|  | 75% | 467.5 | 9.1 | 92.5 | 0.20 | 99.5 | 72.2 | 150.3 | 0.92 | 2.48 | 3736.2 | 493.8 | 169.6 | 0.39 | 0.96 |
|  | *N* | *21* | *20* | *21* | *21* | *21* | *21* | *21* | *21* | *21* | *21* | *21* | *21* | *21* | *21* |
| Parietal cortex | 25% | 510.2 | 14.9 | 83.6 | 0.17 | 24.8 | 8.4 | 11.3 | 0.32 | 0.34 | 3207.9 | 278.6 | 103.5 | 0.32 | 0.08 |
|  | 50% | 549.6 | 21.7 | 104.2 | 0.19 | 30.4 | 12.7 | 17.0 | 0.44 | 0.56 | 3659.6 | 357.4 | 128.7 | 0.34 | 0.14 |
|  | 75% | 603.7 | 33.8 | 123.1 | 0.22 | 35.5 | 14.9 | 24.3 | 0.51 | 0.89 | 4043.5 | 408.9 | 139.9 | 0.39 | 0.19 |
|  | *N* | *21* | *19* | *21* | *21* | *21* | *21* | *21* | *21* | *21* | *21* | *21* | *21* | *21* | *21* |
| Occipital cortex | 25% | 330.8 | 8.3 | 47.3 | 0.13 | 16.8 | 2.9 | 6.1 | 0.11 | 0.27 | 3224.9 | 138.5 | 96.4 | 0.51 | 0.06 |
|  | 50% | 356.3 | 11.1 | 55.1 | 0.15 | 19.8 | 6.2 | 11.4 | 0.24 | 0.51 | 3588.1 | 169.7 | 114.5 | 0.62 | 0.10 |
|  | 75% | 412.3 | 14.0 | 66.9 | 0.18 | 26.6 | 7.6 | 13.4 | 0.37 | 0.74 | 3923.0 | 198.4 | 122.0 | 0.78 | 0.12 |
|  | *N* | *21* | *21* | *21* | *21* | *21* | *20* | *19* | *20* | *19* | *21* | *21* | *21* | *21* | *19* |
| Hippocampus | 25% | 390.9 | 4.8 | 35.3 | 0.09 | 34.5 | 15.6 | 44.9 | 0.39 | 0.99 | 3106.4 | 466.4 | 346.9 | 0.70 | 0.11 |
|  | 50% | 428.8 | 6.1 | 48.7 | 0.10 | 42.3 | 22.3 | 50.7 | 0.44 | 1.11 | 3492.0 | 518.4 | 404.9 | 0.80 | 0.12 |
|  | 75% | 468.8 | 9.9 | 74.3 | 0.16 | 51.7 | 24.4 | 54.6 | 0.55 | 1.32 | 4218.8 | 551.5 | 454.8 | 0.85 | 0.15 |
|  | *N* | *21* | *14* | *21* | *21* | *21* | *21* | *21* | *21* | *21* | *21* | *21* | *21* | *21* | *21* |
| Striatum | 25% | 96.6 | 4.0 | 33.0 | 0.27 | 10299.0 | 936.5 | 1252.5 | 0.08 | 0.10 | 3064.6 | 323.7 | 289.4 | 0.83 | 3.91 |
|  | 50% | 115.6 | 9.8 | 38.8 | 0.33 | 12237.7 | 1089.2 | 1413.2 | 0.09 | 0.12 | 3370.9 | 357.2 | 303.7 | 0.87 | 4.49 |
|  | 75% | 129.4 | 14.5 | 54.0 | 0.45 | 13311.1 | 1247.3 | 1576.5 | 0.10 | 0.13 | 3896.1 | 383.0 | 341.3 | 0.97 | 4.90 |
|  | *N* | *21* | *16* | *21* | *21* | *21* | *21* | *21* | *21* | *21* | *21* | *21* | *21* | *21* | *21* |
| (Hypo)thalamus | 25% | 847.0 | 3.8 | 76.1 | 0.08 | 209.3 | 104.7 | 185.2 | 0.42 | 0.72 | 3381.8 | 605.0 | 424.7 | 0.66 | 0.38 |
|  | 50% | 942.8 | 8.5 | 86.1 | 0.09 | 253.1 | 121.5 | 199.9 | 0.48 | 0.81 | 3670.0 | 629.5 | 443.9 | 0.69 | 0.45 |
|  | 75% | 1012.9 | 13.0 | 95.4 | 0.11 | 281.0 | 138.2 | 224.8 | 0.58 | 1.02 | 4007.2 | 666.7 | 510.1 | 0.79 | 0.50 |
|  | *N* | *21* | *21* | *21* | *21* | *21* | *21* | *21* | *21* | *21* | *21* | *21* | *21* | *21* | *21* |
| Brainstem | 25% | 508.3 | 3.7 | 60.5 | 0.12 | 36.4 | 28.5 | 40.1 | 0.69 | 0.98 | 2518.2 | 428.7 | 408.9 | 0.84 | 0.09 |
|  | 50% | 575.6 | 5.5 | 72.9 | 0.12 | 41.2 | 35.6 | 50.3 | 0.75 | 1.23 | 2785.6 | 479.7 | 470.4 | 1.01 | 0.11 |
|  | 75% | 601.8 | 7.4 | 82.4 | 0.14 | 48.7 | 38.8 | 65.2 | 0.91 | 1.50 | 3023.6 | 520.8 | 519.4 | 1.17 | 0.13 |
|  | *N* | *21* | *17* | *21* | *21* | *21* | *21* | *21* | *21* | *21* | *21* | *21* | *21* | *21* | *21* |
| Cerebellum | 25% | 264.6 | 2.3 | 27.6 | 0.08 | 10.8 | 1.5 | 7.6 | 0.11 | 0.55 | 3359.2 | 70.4 | 80.1 | 1.01 | 0.08 |
|  | 50% | 301.2 | 4.8 | 29.9 | 0.10 | 11.7 | 3.1 | 10.3 | 0.23 | 0.70 | 3550.7 | 90.5 | 98.6 | 1.16 | 0.10 |
|  | 75% | 353.6 | 7.7 | 35.9 | 0.14 | 18.8 | 5.5 | 13.2 | 0.51 | 1.09 | 4177.9 | 97.3 | 108.1 | 1.44 | 0.12 |
|  | *N* | *21* | *18* | *21* | *21* | *21* | *21* | *21* | *21* | *21* | *21* | *21* | *21* | *21* | *21* |
| Olfactory bulb | 25% | 246.8 | 5.3 | 40.6 | 0.15 | 163.3 | 58.6 | 127.5 | 0.36 | 0.79 | 3362.8 | 287.0 | 121.8 | 0.38 | 0.92 |
|  | 50% | 310.3 | 6.6 | 49.7 | 0.16 | 199.7 | 79.7 | 170.7 | 0.43 | 0.87 | 3552.6 | 323.5 | 139.7 | 0.43 | 1.14 |
|  | 75% | 325.7 | 7.9 | 54.0 | 0.19 | 225.3 | 106.7 | 199.8 | 0.49 | 1.00 | 3882.2 | 355.2 | 164.0 | 0.51 | 1.49 |
|  | *N* | *21* | *15* | *21* | *21* | *21* | *21* | *21* | *21* | *21* | *21* | *21* | *21* | *21* | *21* |

Monoamines, metabolites and ratios are expressed as median (50%) with the interquartile range (25%-75%). The number of samples in which a specific compound was detected is provided as well. Concentrations (ng/ml) are rounded off to one decimal, the ratios to two decimals. 5-HIAA, 5-hydroxyindoleacetic acid; 5-HT, serotonin; A, adrenaline; DA, dopamine; DOPAC, 3-4-dihydroxyphenylacetic acid; HVA, homovanillic acid; MHPG, 3-methoxy-4-hydroxyphenylglycol; NA, noradrenaline.

**Table S2: Concentrations of monoaminergic compounds and ratios in young WT_Ts65Dn_ mice**

|  |  | **(nor)adrenergic system** | | | | **dopaminergic system** | | | | | **serotonergic system** | | | | |
| --- | --- | --- | --- | --- | --- | --- | --- | --- | --- | --- | --- | --- | --- | --- | --- |
|  |  | **NA**  **(ng/ml)** | **A**  **(ng/ml)** | **MHPG**  **(ng/ml)** | **MHPG**  **/NA** | **DA**  **(ng/ml)** | **DOPAC**  **(ng/ml)** | **HVA**  **(ng/ml)** | **DOPAC**  **/DA** | **HVA**  **/DA** | **TRP**  **(ng/ml)** | **5-HT**  **(ng/ml)** | **5-HIAA**  **(ng/ml)** | **5HIAA**  **/5HT** | **HVA**  **/5HIAA** |
| Frontal cortex | 25% | 346.8 | 16.2 | 74.0 | 0.16 | 31.5 | 20.6 | 55.9 | 0.54 | 1.43 | 2763.2 | 541.2 | 136.9 | 0.22 | 0.38 |
|  | 50% | 405.6 | 24.7 | 82.5 | 0.20 | 35.2 | 25.1 | 62.1 | 0.64 | 1.66 | 3317.2 | 591.9 | 154.2 | 0.26 | 0.42 |
|  | 75% | 511.6 | 31.0 | 94.8 | 0.24 | 52.0 | 31.0 | 71.9 | 0.84 | 2.30 | 4074.8 | 633.5 | 161.4 | 0.28 | 0.48 |
|  | *N* | *18* | *18* | *18* | *18* | *18* | *18* | *18* | *18* | *18* | *18* | *18* | *18* | *18* | *18* |
| Temporal cortex | 25% | 379.8 | 3.8 | 62.7 | 0.14 | 32.6 | 31.1 | 74.7 | 0.61 | 1.33 | 3081.5 | 383.3 | 151.3 | 0.32 | 0.50 |
|  | 50% | 432.1 | 5.9 | 75.3 | 0.18 | 52.4 | 40.6 | 110.0 | 0.79 | 1.87 | 3471.4 | 474.8 | 185.0 | 0.36 | 0.62 |
|  | 75% | 461.5 | 8.7 | 87.5 | 0.21 | 111.5 | 70.5 | 140.1 | 1.03 | 2.64 | 3850.9 | 600.3 | 200.8 | 0.45 | 0.76 |
|  | *N* | *18* | *17* | *18* | *18* | *18* | *18* | *18* | *18* | *18* | *18* | *18* | *18* | *18* | *18* |
| Parietal cortex | 25% | 442.8 | 21.2 | 83.1 | 0.17 | 27.4 | 10.3 | 17.4 | 0.24 | 0.40 | 3131.5 | 314.2 | 126.7 | 0.33 | 0.12 |
|  | 50% | 502.5 | 23.4 | 101.1 | 0.18 | 37.1 | 12.8 | 21.2 | 0.38 | 0.56 | 3676.1 | 379.4 | 148.6 | 0.40 | 0.16 |
|  | 75% | 599.6 | 26.7 | 114.4 | 0.21 | 48.3 | 15.8 | 31.1 | 0.45 | 0.96 | 4285.2 | 443.4 | 160.7 | 0.46 | 0.18 |
|  | *N* | *18* | *18* | *18* | *18* | *18* | *18* | *17* | *18* | *17* | *18* | *18* | *18* | *18* | *17* |
| Occipital cortex | 25% | 320.1 | 10.7 | 46.8 | 0.13 | 15.2 | 3.3 | 8.1 | 0.20 | 0.42 | 3236.6 | 165.7 | 123.0 | 0.49 | 0.07 |
|  | 50% | 331.3 | 12.1 | 53.0 | 0.15 | 19.4 | 6.2 | 11.5 | 0.23 | 0.64 | 3584.6 | 221.0 | 132.4 | 0.60 | 0.09 |
|  | 75% | 389.5 | 13.9 | 69.6 | 0.19 | 25.5 | 9.7 | 15.6 | 0.44 | 0.85 | 3926.8 | 277.1 | 142.3 | 0.76 | 0.10 |
|  | *N* | *18* | *18* | *18* | *18* | *18* | *15* | *17* | *15* | *17* | *18* | *18* | *18* | *18* | *17* |
| Hippocampus | 25% | 383.5 | 5.1 | 37.9 | 0.09 | 37.0 | 17.4 | 46.3 | 0.35 | 1.02 | 3162.6 | 532.1 | 397.6 | 0.74 | 0.09 |
|  | 50% | 460.7 | 7.1 | 71.0 | 0.13 | 42.8 | 21.3 | 52.2 | 0.48 | 1.15 | 3566.9 | 574.2 | 473.4 | 0.80 | 0.11 |
|  | 75% | 560.3 | 12.0 | 81.3 | 0.17 | 51.3 | 27.9 | 61.6 | 0.63 | 1.38 | 4454.1 | 683.3 | 561.3 | 0.88 | 0.12 |
|  | *N* | *18* | *13* | *18* | *18* | *18* | *18* | *18* | *18* | *18* | *18* | *18* | *18* | *18* | *18* |
| Striatum | 25% | 94.4 | 3.7 | 36.1 | 0.25 | 9842.3 | 967.6 | 1191.3 | 0.09 | 0.11 | 2932.3 | 356.8 | 303.9 | 0.76 | 3.79 |
|  | 50% | 132.6 | 8.7 | 43.7 | 0.32 | 11849.4 | 1188.0 | 1391.1 | 0.10 | 0.12 | 3536.3 | 387.8 | 330.2 | 0.86 | 4.45 |
|  | 75% | 160.9 | 13.7 | 49.6 | 0.49 | 14123.5 | 1370.4 | 1598.0 | 0.11 | 0.12 | 3997.4 | 438.5 | 360.0 | 0.95 | 4.76 |
|  | *N* | *18* | *16* | *18* | *18* | *18* | *18* | *18* | *18* | *18* | *18* | *18* | *18* | *18* | *18* |
| (Hypo)thalamus | 25% | 830.6 | 8.0 | 78.0 | 0.08 | 231.6 | 129.6 | 201.9 | 0.36 | 0.52 | 3529.3 | 625.2 | 445.5 | 0.60 | 0.41 |
|  | 50% | 960.9 | 14.8 | 84.6 | 0.10 | 324.4 | 147.7 | 232.5 | 0.44 | 0.78 | 3746.7 | 687.6 | 459.6 | 0.66 | 0.51 |
|  | 75% | 1117.2 | 19.7 | 101.8 | 0.11 | 402.6 | 163.0 | 264.7 | 0.53 | 0.89 | 4100.1 | 744.5 | 502.5 | 0.83 | 0.55 |
|  | *N* | *18* | *18* | *18* | *18* | *18* | *18* | *18* | *18* | *18* | *18* | *18* | *18* | *18* | *18* |
| Brainstem | 25% | 504.5 | 2.6 | 65.0 | 0.11 | 36.9 | 28.5 | 40.7 | 0.72 | 0.99 | 2456.8 | 466.0 | 390.7 | 0.80 | 0.09 |
|  | 50% | 562.0 | 5.3 | 66.8 | 0.12 | 40.0 | 35.1 | 48.5 | 0.83 | 1.24 | 2710.7 | 504.2 | 477.4 | 1.00 | 0.11 |
|  | 75% | 643.2 | 7.4 | 75.9 | 0.13 | 47.3 | 44.1 | 60.7 | 0.98 | 1.40 | 3142.2 | 561.5 | 525.4 | 1.07 | 0.12 |
|  | *N* | *18* | *15* | *18* | *18* | *18* | *18* | *18* | *18* | *18* | *18* | *18* | *18* | *18* | *18* |
| Cerebellum | 25% | 304.2 | 3.6 | 26.6 | 0.08 | 11.1 | 1.7 | 7.0 | 0.12 | 0.46 | 3095.2 | 74.2 | 81.1 | 0.98 | 0.08 |
|  | 50% | 326.1 | 4.5 | 33.0 | 0.10 | 14.3 | 3.5 | 10.1 | 0.22 | 0.74 | 3446.4 | 84.6 | 92.5 | 1.08 | 0.11 |
|  | 75% | 361.3 | 7.3 | 41.7 | 0.13 | 17.2 | 5.7 | 16.1 | 0.44 | 1.06 | 4167.6 | 100.9 | 109.3 | 1.32 | 0.16 |
|  | *N* | *18* | *15* | *18* | *18* | *18* | *17* | *18* | *17* | *18* | *18* | *18* | *18* | *18* | *18* |
| Olfactory bulb | 25% | 252.7 | 6.1 | 32.7 | 0.10 | 200.6 | 68.7 | 142.6 | 0.32 | 0.62 | 3249.7 | 237.4 | 119.7 | 0.41 | 1.09 |
|  | 50% | 289.7 | 6.5 | 36.1 | 0.12 | 233.9 | 96.9 | 175.2 | 0.38 | 0.74 | 3472.2 | 265.7 | 130.2 | 0.44 | 1.38 |
|  | 75% | 340.9 | 9.6 | 45.2 | 0.16 | 298.5 | 114.5 | 221.0 | 0.43 | 0.83 | 4051.3 | 320.6 | 142.0 | 0.54 | 1.67 |
|  | *N* | *17* | *9* | *17* | *17* | *17* | *17* | *17* | *17* | *17* | *17* | *17* | *17* | *17* | *17* |

Monoamines, metabolites and ratios are expressed as median (50%) with the interquartile range (25%-75%). The number of samples in which a specific compound was detected is provided as well. Concentrations (ng/ml) are rounded off to one decimal, the ratios to two decimals. 5-HIAA, 5-hydroxyindoleacetic acid; 5-HT, serotonin; A, adrenaline; DA, dopamine; DOPAC, 3-4-dihydroxyphenylacetic acid; HVA, homovanillic acid; MHPG, 3-methoxy-4-hydroxyphenylglycol; NA, noradrenaline.

**Table S3: Concentrations of monoaminergic compounds and ratios in aged Ts65Dn mice**

|  |  |  |  |  |  |  |  |  |  |  |  |  |  |  |  |
| --- | --- | --- | --- | --- | --- | --- | --- | --- | --- | --- | --- | --- | --- | --- | --- |
|  |  | **NA**  **(ng/ml)** | **A**  **(ng/ml)** | **MHPG**  **(ng/ml)** | **MHPG**  **/NA** | **DA**  **(ng/ml)** | **DOPAC**  **(ng/ml)** | **HVA**  **(ng/ml)** | **DOPAC**  **/DA** | **HVA**  **/DA** | **TRP**  **(ng/ml)** | **5-HT**  **(ng/ml)** | **5-HIAA**  **(ng/ml)** | **5HIAA**  **/5HT** | **HVA**  **/5HIAA** |
| Frontal cortex | 25% | 378.2 | 3.3 | 61.3 | 0.13 | 26.1 | 14.2 | 35.5 | 0.49 | 0.79 | 2873.5 | 439.4 | 146.1 | 0.23 | 0.27 |
|  | 50% | 430.8 | 8.9 | 63.6 | 0.14 | 32.5 | 18.0 | 54.7 | 0.61 | 1.72 | 3321.3 | 546.3 | 160.8 | 0.32 | 0.31 |
|  | 75% | 468.5 | 15.0 | 70.6 | 0.19 | 46.9 | 29.9 | 89.7 | 0.83 | 2.10 | 3502.6 | 607.0 | 184.2 | 0.42 | 0.36 |
|  | *N* | *11* | *11* | *11* | *11* | *11* | *11* | *11* | *11* | *11* | *11* | *11* | *11* | *11* | *11* |
| Temporal cortex | 25% | 332.3 | 13.7 | 71.2 | 0.17 | 114.7 | 69.3 | 87.9 | 0.31 | 0.50 | 3311.1 | 440.8 | 216.2 | 0.43 | 0.31 |
|  | 50% | 356.2 | 16.2 | 87.8 | 0.20 | 180.7 | 79.3 | 114.2 | 0.46 | 0.62 | 3928.2 | 539.7 | 231.5 | 0.46 | 0.53 |
|  | 75% | 488.6 | 30.8 | 93.9 | 0.27 | 265.3 | 108.2 | 143.7 | 0.69 | 1.08 | 4907.5 | 575.5 | 281.1 | 0.53 | 0.62 |
|  | *N* | *11* | *5* | *11* | *11* | *11* | *11* | *11* | *11* | *11* | *11* | *11* | *11* | *11* | *11* |
| Parietal cortex | 25% | 460.7 | 6.4 | 44.2 | 0.08 | 20.2 | 5.2 | 9.2 | 0.19 | 0.47 | 3249.9 | 268.4 | 145.8 | 0.36 | 0.05 |
|  | 50% | 548.5 | 11.7 | 59.2 | 0.10 | 27.4 | 9.1 | 15.5 | 0.26 | 0.55 | 3412.1 | 341.1 | 170.6 | 0.51 | 0.09 |
|  | 75% | 628.3 | 12.4 | 79.9 | 0.14 | 41.5 | 12.3 | 27.4 | 0.46 | 1.06 | 3794.5 | 399.4 | 210.5 | 0.70 | 0.16 |
|  | *N* | *11* | *7* | *11* | *11* | *11* | *11* | *11* | *11* | *11* | *11* | *11* | *11* | *11* | *11* |
| Occipital cortex | 25% | 275.3 | 0.5 | 22.8 | 0.08 | 13.9 | 4.1 | 3.1 | 0.06 | 0.13 | 3219.0 | 180.7 | 113.8 | 0.56 | 0.03 |
|  | 50% | 354.1 | 8.8 | 43.3 | 0.12 | 36.9 | 5.3 | 9.2 | 0.13 | 0.19 | 3336.6 | 204.9 | 127.6 | 0.62 | 0.05 |
|  | 75% | 434.8 | 14.7 | 57.4 | 0.14 | 101.7 | 7.1 | 10.8 | 0.32 | 0.29 | 3829.7 | 238.3 | 167.8 | 0.68 | 0.08 |
|  | *N* | *11* | *9* | *11* | *11* | *11* | *11* | *7* | *11* | *7* | *11* | *11* | *11* | *11* | *7* |
| Hippocampus | 25% | 359.6 | 2.2 | 31.8 | 0.09 | 29.1 | 17.3 | 31.5 | 0.39 | 0.92 | 3117.5 | 435.4 | 373.5 | 0.71 | 0.08 |
|  | 50% | 409.6 | 3.4 | 45.5 | 0.12 | 38.2 | 21.0 | 39.6 | 0.53 | 1.06 | 3326.0 | 528.7 | 434.2 | 0.86 | 0.10 |
|  | 75% | 451.8 | 5.8 | 62.8 | 0.16 | 44.0 | 26.3 | 45.1 | 0.66 | 1.27 | 3855.0 | 574.9 | 480.0 | 0.93 | 0.13 |
|  | *N* | *11* | *11* | *11* | *11* | *11* | *11* | *11* | *11* | *11* | *11* | *11* | *11* | *11* | *11* |
| Striatum | 25% | 130.2 | 6.1 | 35.5 | 0.17 | 7139.0 | 731.3 | 908.8 | 0.09 | 0.10 | 3362.3 | 385.9 | 282.7 | 0.64 | 2.64 |
|  | 50% | 183.9 | 12.5 | 41.3 | 0.24 | 8142.8 | 853.4 | 1114.1 | 0.10 | 0.12 | 3378.7 | 430.3 | 303.1 | 0.75 | 3.04 |
|  | 75% | 220.3 | 19.4 | 45.9 | 0.27 | 11305.9 | 1120.9 | 1126.1 | 0.12 | 0.14 | 3746.8 | 543.2 | 369.3 | 0.82 | 4.28 |
|  | *N* | *11* | *7* | *11* | *11* | *11* | *11* | *11* | *11* | *11* | *11* | *11* | *11* | *11* | *11* |
| (Hypo)thalamus | 25% | 582.7 | 1.6 | 70.7 | 0.07 | 186.1 | 81.4 | 154.6 | 0.36 | 0.58 | 3382.9 | 530.0 | 466.6 | 0.77 | 0.32 |
|  | 50% | 766.4 | 3.3 | 80.0 | 0.10 | 232.1 | 116.3 | 172.2 | 0.51 | 0.76 | 3485.3 | 595.3 | 527.1 | 0.88 | 0.33 |
|  | 75% | 920.3 | 4.2 | 86.0 | 0.15 | 315.8 | 146.7 | 216.0 | 0.65 | 0.91 | 4201.7 | 749.9 | 617.8 | 0.92 | 0.35 |
|  | *N* | *11* | *11* | *11* | *11* | *11* | *11* | *11* | *11* | *11* | *11* | *11* | *11* | *11* | *11* |
| Brainstem | 25% | 537.9 | 1.6 | 69.8 | 0.13 | 34.2 | 27.8 | 46.3 | 0.75 | 1.11 | 2519.3 | 432.9 | 438.1 | 0.90 | 0.09 |
|  | 50% | 550.0 | 2.2 | 71.1 | 0.15 | 41.6 | 35.3 | 47.1 | 0.88 | 1.30 | 2635.2 | 484.9 | 464.4 | 0.98 | 0.10 |
|  | 75% | 584.6 | 2.5 | 85.5 | 0.16 | 44.1 | 41.6 | 56.7 | 0.97 | 1.43 | 2963.2 | 512.8 | 525.1 | 1.20 | 0.13 |
|  | *N* | *11* | *7* | *11* | *11* | *11* | *11* | *11* | *11* | *11* | *11* | *11* | *11* | *11* | *11* |
| Cerebellum | 25% | 244.7 | 2.4 | 15.3 | 0.06 | 11.0 | 9.5 | 9.3 | 0.79 | 0.81 | 3420.5 | 82.5 | 98.4 | 0.76 | 0.09 |
|  | 50% | 293.8 | 3.5 | 22.1 | 0.07 | 12.0 | 11.9 | 10.9 | 0.96 | 1.07 | 3554.8 | 106.4 | 123.0 | 1.13 | 0.10 |
|  | 75% | 367.8 | 10.4 | 42.1 | 0.10 | 13.8 | 14.6 | 16.5 | 1.18 | 1.17 | 3739.1 | 146.8 | 138.5 | 1.55 | 0.12 |
|  | *N* | *11* | *11* | *11* | *11* | *11* | *11* | *11* | *11* | *11* | *11* | *11* | *11* | *11* | *11* |
| Olfactory bulb | 25% | 241.3 | 5.3 | 53.2 | 0.19 | 164.4 | 83.6 | 153.2 | 0.46 | 0.78 | 3476.4 | 293.3 | 132.0 | 0.43 | 0.70 |
|  | 50% | 282.6 | 7.2 | 62.8 | 0.20 | 223.0 | 113.3 | 192.9 | 0.51 | 0.89 | 4535.9 | 346.8 | 162.2 | 0.47 | 1.23 |
|  | 75% | 324.8 | 10.6 | 71.8 | 0.30 | 245.5 | 132.3 | 205.3 | 0.64 | 0.93 | 5485.3 | 378.6 | 202.7 | 0.58 | 1.54 |
|  | *N* | *11* | *6* | *11* | *11* | *11* | *11* | *11* | *11* | *11* | *11* | *11* | *11* | *11* | *11* |

Monoamines, metabolites and ratios are expressed as median (50%) with the interquartile range (25%-75%). The number of samples in which a specific compound was detected is provided as well. Concentrations (ng/ml) are rounded off to one decimal, the ratios to two decimals. 5-HIAA, 5-hydroxyindoleacetic acid; 5-HT, serotonin; A, adrenaline; DA, dopamine; DOPAC, 3-4-dihydroxyphenylacetic acid; HVA, homovanillic acid; MHPG, 3-methoxy-4-hydroxyphenylglycol; NA, noradrenaline.

**Table S4: Concentrations of monoaminergic compounds and ratios in aged WT_Ts65Dn_ mice**

|  |  |  |  |  |  |  |  |  |  |  |  |  |  |  |  |
| --- | --- | --- | --- | --- | --- | --- | --- | --- | --- | --- | --- | --- | --- | --- | --- |
|  |  | **NA**  **(ng/ml)** | **A**  **(ng/ml)** | **MHPG**  **(ng/ml)** | **MHPG**  **/NA** | **DA**  **(ng/ml)** | **DOPAC**  **(ng/ml)** | **HVA**  **(ng/ml)** | **DOPAC**  **/DA** | **HVA**  **/DA** | **TRP**  **(ng/ml)** | **5-HT**  **(ng/ml)** | **5-HIAA**  **(ng/ml)** | **5HIAA**  **/5HT** | **HVA**  **/5HIAA** |
| Frontal cortex | 25% | 408.3 | 1.1 | 73.7 | 0.17 | 20.5 | 19.3 | 55.1 | 0.82 | 2.21 | 3152.2 | 437.6 | 164.7 | 0.30 | 0.30 |
|  | 50% | 435.9 | 2.9 | 78.9 | 0.19 | 24.9 | 26.5 | 66.5 | 0.94 | 2.43 | 3464.6 | 485.3 | 179.6 | 0.38 | 0.36 |
|  | 75% | 446.7 | 8.6 | 85.4 | 0.20 | 40.7 | 33.5 | 79.1 | 1.14 | 2.84 | 3776.6 | 544.4 | 189.2 | 0.42 | 0.47 |
|  | *N* | *10* | *10* | *10* | *10* | *10* | *10* | *10* | *10* | *10* | *10* | *10* | *10* | *10* | *10* |
| Temporal cortex | 25% | 335.1 | - | 84.1 | 0.23 | 128.5 | 52.9 | 56.3 | 0.26 | 0.23 | 4676.7 | 489.2 | 258.5 | 0.43 | 0.20 |
|  | 50% | 357.5 | - | 92.4 | 0.26 | 225.4 | 75.3 | 82.8 | 0.35 | 0.30 | 5354.4 | 611.2 | 289.6 | 0.45 | 0.32 |
|  | 75% | 386.7 | - | 95.7 | 0.27 | 436.9 | 146.8 | 115.7 | 0.47 | 0.43 | 6314.0 | 701.1 | 306.8 | 0.52 | 0.40 |
|  | *N* | *10* | *0* | *10* | *10* | *10* | *10* | *10* | *10* | *10* | *10* | *10* | *10* | *10* | *10* |
| Parietal cortex | 25% | 489.7 | 9.6 | 39.1 | 0.08 | 18.4 | 13.5 | 21.9 | 0.48 | 0.76 | 3228.0 | 253.5 | 135.6 | 0.47 | 0.11 |
|  | 50% | 540.3 | 10.2 | 42.8 | 0.08 | 29.3 | 17.5 | 28.4 | 0.60 | 1.08 | 3543.6 | 298.4 | 167.5 | 0.53 | 0.17 |
|  | 75% | 557.5 | 12.6 | 47.1 | 0.09 | 34.4 | 20.1 | 40.7 | 0.81 | 1.46 | 3898.5 | 333.5 | 194.5 | 0.72 | 0.27 |
|  | *N* | *10* | *10* | *10* | *10* | *10* | *10* | *10* | *10* | *10* | *10* | *10* | *10* | *10* | *10* |
| Occipital cortex | 25% | 292.5 | 0.7 | 27.7 | 0.08 | 12.1 | 2.4 | 0.5 | 0.18 | 0.02 | 3217.2 | 154.1 | 102.4 | 0.57 | 0.00 |
|  | 50% | 334.0 | 1.1 | 30.7 | 0.10 | 15.8 | 3.5 | 0.9 | 0.21 | 0.07 | 3456.4 | 188.3 | 124.8 | 0.64 | 0.01 |
|  | 75% | 398.3 | 1.6 | 38.6 | 0.11 | 27.5 | 9.1 | 4.2 | 0.33 | 0.25 | 3698.5 | 228.2 | 175.7 | 0.90 | 0.02 |
|  | *N* | *10* | *8* | *10* | *10* | *10* | *10* | *9* | *10* | *9* | *10* | *10* | *10* | *10* | *9* |
| Hippocampus | 25% | 435.4 | 3.1 | 43.2 | 0.09 | 43.2 | 22.1 | 39.1 | 0.37 | 0.67 | 3452.0 | 470.4 | 444.1 | 0.87 | 0.08 |
|  | 50% | 454.8 | 4.1 | 56.7 | 0.13 | 52.1 | 34.1 | 51.7 | 0.55 | 0.87 | 3582.8 | 525.4 | 495.6 | 0.92 | 0.10 |
|  | 75% | 503.4 | 5.8 | 62.6 | 0.14 | 83.6 | 39.2 | 57.7 | 0.62 | 1.04 | 4011.8 | 636.4 | 542.1 | 0.99 | 0.12 |
|  | *N* | *10* | *9* | *10* | *10* | *10* | *10* | *10* | *10* | *10* | *10* | *10* | *10* | *10* | *10* |
| Striatum | 25% | 164.8 | 1.4 | 36.5 | 0.16 | 7656.5 | 769.3 | 855.2 | 0.10 | 0.11 | 3529.6 | 448.9 | 282.0 | 0.58 | 2.76 |
|  | 50% | 217.6 | 3.3 | 39.5 | 0.22 | 8461.0 | 879.9 | 897.7 | 0.11 | 0.11 | 3672.4 | 469.9 | 302.6 | 0.63 | 3.03 |
|  | 75% | 241.7 | - | 46.1 | 0.25 | 8961.6 | 1094.5 | 1075.6 | 0.12 | 0.13 | 3905.0 | 532.1 | 333.4 | 0.71 | 3.24 |
|  | *N* | *10* | *3* | *10* | *10* | *10* | *10* | *10* | *10* | *10* | *10* | *10* | *10* | *10* | *10* |
| (Hypo)thalamus | 25% | 709.5 | 2.4 | 75.1 | 0.09 | 197.2 | 102.7 | 160.9 | 0.43 | 0.60 | 3497.3 | 592.8 | 483.7 | 0.72 | 0.30 |
|  | 50% | 828.3 | 3.3 | 79.8 | 0.10 | 263.0 | 120.6 | 175.5 | 0.48 | 0.63 | 3847.1 | 645.0 | 536.3 | 0.82 | 0.32 |
|  | 75% | 974.3 | 5.8 | 99.7 | 0.11 | 320.2 | 168.9 | 190.4 | 0.54 | 0.86 | 4291.5 | 736.0 | 606.0 | 0.91 | 0.34 |
|  | *N* | *10* | *10* | *10* | *10* | *10* | *10* | *10* | *10* | *10* | *10* | *10* | *10* | *10* | *10* |
| Brainstem | 25% | 500.2 | 1.3 | 77.4 | 0.14 | 37.4 | 35.1 | 40.5 | 0.85 | 0.95 | 2822.9 | 430.3 | 444.8 | 0.90 | 0.09 |
|  | 50% | 567.3 | 1.5 | 83.4 | 0.15 | 39.8 | 41.1 | 44.1 | 0.97 | 1.14 | 3041.9 | 447.6 | 480.2 | 0.97 | 0.09 |
|  | 75% | 609.9 | - | 95.0 | 0.17 | 46.2 | 45.3 | 48.8 | 1.14 | 1.30 | 3091.8 | 519.1 | 504.5 | 1.13 | 0.10 |
|  | *N* | *10* | *3* | *10* | *10* | *10* | *10* | *10* | *10* | *10* | *10* | *10* | *10* | *10* | *10* |
| Cerebellum | 25% | 250.4 | 2.0 | 13.5 | 0.04 | 10.2 | 14.1 | 12.9 | 1.11 | 1.07 | 3412.0 | 72.4 | 121.8 | 1.32 | 0.10 |
|  | 50% | 332.4 | 4.4 | 19.3 | 0.06 | 12.3 | 15.2 | 14.1 | 1.31 | 1.20 | 3774.6 | 92.0 | 131.7 | 1.46 | 0.11 |
|  | 75% | 435.8 | 8.7 | 27.7 | 0.09 | 15.1 | 19.0 | 16.3 | 1.49 | 1.34 | 3994.3 | 108.2 | 146.0 | 1.82 | 0.13 |
|  | *N* | *10* | *9* | *10* | *10* | *10* | *10* | *10* | *10* | *10* | *10* | *10* | *10* | *10* | *10* |
| Olfactory bulb | 25% | 253.8 | 1.2 | 66.0 | 0.21 | 237.8 | 108.4 | 187.0 | 0.44 | 0.72 | 4380.1 | 265.3 | 114.7 | 0.39 | 1.44 |
|  | 50% | 301.9 | 4.4 | 74.1 | 0.25 | 270.2 | 136.0 | 222.1 | 0.52 | 0.80 | 5654.0 | 293.7 | 132.7 | 0.44 | 1.65 |
|  | 75% | 340.2 | - | 82.1 | 0.31 | 309.4 | 163.4 | 250.8 | 0.57 | 0.89 | 6278.5 | 307.4 | 140.6 | 0.48 | 2.12 |
|  | *N* | *10* | *2* | *10* | *10* | *10* | *10* | *10* | *10* | *10* | *10* | *10* | *10* | *10* | *10* |

Monoamines, metabolites and ratios are expressed as median (50%) with the interquartile range (25%-75%). The number of samples in which a specific compound was detected is provided as well. Concentrations (ng/ml) are rounded off to one decimal, the ratios to two decimals. 5-HIAA, 5-hydroxyindoleacetic acid; 5-HT, serotonin; A, adrenaline; DA, dopamine; DOPAC, 3-4-dihydroxyphenylacetic acid; HVA, homovanillic acid; MHPG, 3-methoxy-4-hydroxyphenylglycol; NA, noradrenaline.

**Table S5: Concentrations of monoaminergic compounds and ratios in young Dp1Tyb mice**

|  |  | **(nor)adrenergic system** | | | | **dopaminergic system** | | | | | **serotonergic system** | | | | |
| --- | --- | --- | --- | --- | --- | --- | --- | --- | --- | --- | --- | --- | --- | --- | --- |
|  |  | **NA**  **(ng/ml)** | **A**  **(ng/ml)** | **MHPG**  **(ng/ml)** | **MHPG**  **/NA** | **DA**  **(ng/ml)** | **DOPAC**  **(ng/ml)** | **HVA**  **(ng/ml)** | **DOPAC**  **/DA** | **HVA**  **/DA** | **TRP**  **(ng/ml)** | **5-HT**  **(ng/ml)** | **5-HIAA**  **(ng/ml)** | **5HIAA**  **/5HT** | **HVA**  **/5HIAA** |
| Frontal cortex | 25% | 571.1 | 10.3 | 75.4 | 0.12 | 40.9 | 21.0 | 72.3 | 0.37 | 1.43 | 3463.8 | 383.4 | 159.0 | 0.36 | 0.41 |
|  | 50% | 603.3 | 19.0 | 88.0 | 0.15 | 48.1 | 23.3 | 98.1 | 0.53 | 1.93 | 5395.8 | 465.3 | 200.1 | 0.40 | 0.47 |
|  | 75% | 643.7 | 28.9 | 102.7 | 0.18 | 56.8 | 25.6 | 112.1 | 0.61 | 2.32 | 5987.7 | 539.3 | 221.4 | 0.44 | 0.55 |
|  | *N* | *9* | *9* | *9* | *9* | *9* | *9* | *9* | *9* | *9* | *9* | *9* | *9* | *9* | *9* |
| Temporal cortex | 25% | 460.5 | 12.6 | 104.2 | 0.19 | 82.0 | 42.1 | 104.1 | 0.24 | 0.55 | 3901.6 | 356.7 | 223.2 | 0.49 | 0.48 |
|  | 50% | 499.8 | 34.2 | 116.3 | 0.22 | 175.1 | 54.7 | 168.0 | 0.28 | 0.70 | 5958.3 | 417.7 | 240.2 | 0.57 | 0.71 |
|  | 75% | 527.4 | 43.9 | 175.8 | 0.40 | 493.9 | 120.2 | 293.7 | 0.68 | 1.59 | 6539.4 | 503.6 | 315.1 | 0.71 | 0.98 |
|  | *N* | *9* | *9* | *9* | *9* | *9* | *9* | *9* | *9* | *9* | *9* | *9* | *9* | *9* | *9* |
| Parietal cortex | 25% | 666.2 | 13.5 | 86.5 | 0.12 | 38.6 | 11.8 | 48.1 | 0.44 | 1.43 | 3900.0 | 363.5 | 198.1 | 0.49 | 0.22 |
|  | 50% | 681.3 | 15.6 | 93.0 | 0.14 | 42.4 | 21.5 | 73.3 | 0.56 | 1.75 | 6186.7 | 451.6 | 232.4 | 0.54 | 0.29 |
|  | 75% | 736.1 | 23.0 | 106.7 | 0.16 | 46.0 | 25.5 | 87.2 | 0.58 | 1.93 | 7277.5 | 473.6 | 255.4 | 0.58 | 0.40 |
|  | *N* | *9* | *8* | *9* | *9* | *9* | *9* | *9* | *9* | *9* | *9* | *9* | *9* | *9* | *9* |
| Occipital cortex | 25% | 456.8 | 9.0 | 76.2 | 0.15 | 13.7 | 4.6 | 11.4 | 0.16 | 0.46 | 4129.1 | 183.5 | 136.9 | 0.63 | 0.06 |
|  | 50% | 499.8 | 10.6 | 83.2 | 0.18 | 22.0 | 5.7 | 14.5 | 0.25 | 0.56 | 6074.8 | 241.2 | 179.0 | 0.76 | 0.09 |
|  | 75% | 535.4 | 14.8 | 95.9 | 0.19 | 33.0 | 9.7 | 17.9 | 0.78 | 1.49 | 6940.9 | 318.0 | 198.9 | 0.93 | 0.12 |
|  | *N* | *9* | *9* | *9* | *9* | *9* | *9* | *9* | *9* | *9* | *9* | *9* | *9* | *9* | *9* |
| Hippocampus | 25% | 508.5 | 5.3 | 42.2 | 0.08 | 29.9 | 18.4 | 63.5 | 0.39 | 1.44 | 4140.8 | 438.3 | 431.0 | 0.95 | 0.12 |
|  | 50% | 535.3 | 5.9 | 50.2 | 0.09 | 40.2 | 20.3 | 67.7 | 0.53 | 1.79 | 5809.4 | 484.3 | 545.7 | 1.09 | 0.13 |
|  | 75% | 582.1 | - | 54.3 | 0.10 | 47.3 | 25.5 | 88.0 | 0.79 | 2.46 | 7194.7 | 521.7 | 728.6 | 1.41 | 0.15 |
|  | *N* | *9* | *2* | *9* | *9* | *9* | *9* | *9* | *9* | *9* | *9* | *9* | *9* | *9* | *9* |
| Striatum | 25% | 139.3 | 4.0 | 65.8 | 0.32 | 9568.7 | 1236.7 | 1618.5 | 0.12 | 0.14 | 5451.1 | 318.6 | 410.5 | 1.07 | 3.68 |
|  | 50% | 160.1 | 8.8 | 72.0 | 0.40 | 11226.2 | 1368.8 | 1826.5 | 0.13 | 0.17 | 7079.9 | 348.4 | 500.4 | 1.30 | 3.74 |
|  | 75% | 230.4 | 13.1 | 78.4 | 0.54 | 11743.3 | 1707.5 | 2022.2 | 0.16 | 0.19 | 7478.4 | 408.4 | 553.4 | 1.51 | 4.11 |
|  | *N* | *9* | *7* | *9* | *9* | *9* | *9* | *9* | *9* | *9* | *9* | *9* | *9* | *9* | *9* |
| (Hypo)thalamus | 25% | 601.6 | 2.6 | 80.0 | 0.11 | 77.3 | 51.9 | 145.0 | 0.45 | 1.20 | 4163.8 | 390.9 | 563.6 | 1.06 | 0.23 |
|  | 50% | 908.1 | 6.3 | 96.3 | 0.12 | 161.1 | 108.6 | 280.0 | 0.54 | 1.48 | 5928.0 | 499.3 | 630.5 | 1.38 | 0.49 |
|  | 75% | 942.8 | 17.9 | 119.5 | 0.14 | 276.9 | 141.2 | 392.8 | 0.75 | 1.94 | 7186.1 | 566.9 | 756.5 | 1.54 | 0.60 |
|  | *N* | *8* | *8* | *8* | *8* | *8* | *8* | *8* | *8* | *8* | *8* | *8* | *8* | *8* | *8* |
| Brainstem | 25% | 593.9 | 6.7 | 79.6 | 0.13 | 46.0 | 38.8 | 102.3 | 0.64 | 1.44 | 3646.2 | 440.3 | 696.6 | 1.42 | 0.12 |
|  | 50% | 623.9 | 6.7 | 86.3 | 0.14 | 53.3 | 43.0 | 109.7 | 0.81 | 2.11 | 5801.1 | 476.5 | 869.6 | 1.81 | 0.13 |
|  | 75% | 730.3 | 6.7 | 95.1 | 0.14 | 78.2 | 57.2 | 156.2 | 0.92 | 2.58 | 6765.1 | 534.8 | 1032.2 | 2.09 | 0.15 |
|  | *N* | *9* | *1* | *9* | *9* | *9* | *9* | *9* | *9* | *9* | *9* | *9* | *9* | *9* | *9* |
| Cerebellum | 25% | 431.4 | 3.2 | 47.2 | 0.11 | 17.5 | 9.8 | 19.5 | 0.50 | 1.01 | 4058.1 | 104.9 | 127.9 | 1.04 | 0.13 |
|  | 50% | 447.3 | 4.3 | 61.2 | 0.14 | 19.1 | 11.9 | 27.8 | 0.61 | 1.28 | 6532.5 | 136.4 | 182.8 | 1.24 | 0.15 |
|  | 75% | 491.5 | 8.1 | 85.8 | 0.19 | 21.4 | 14.0 | 31.1 | 0.71 | 1.64 | 7538.0 | 183.7 | 210.9 | 1.50 | 0.17 |
|  | *N* | *9* | *7* | *9* | *9* | *9* | *9* | *9* | *9* | *9* | *9* | *9* | *9* | *9* | *9* |
| Olfactory bulb | 25% | 352.1 | 4.8 | 42.7 | 0.12 | 206.9 | 66.7 | 189.4 | 0.28 | 0.73 | 3664.1 | 326.7 | 184.4 | 0.56 | 0.83 |
|  | 50% | 387.2 | 5.9 | 53.1 | 0.13 | 233.6 | 78.6 | 246.6 | 0.34 | 1.04 | 4913.2 | 341.0 | 234.6 | 0.66 | 1.00 |
|  | 75% | 416.7 | 10.2 | 60.3 | 0.14 | 248.5 | 95.8 | 253.0 | 0.44 | 1.22 | 5816.3 | 397.8 | 265.6 | 0.76 | 1.11 |
|  | *N* | *8* | *7* | *8* | *8* | *8* | *8* | *8* | *8* | *8* | *8* | *8* | *8* | *8* | *8* |

Monoamines, metabolites and ratios are expressed as median (50%) with the interquartile range (25%-75%). The number of samples in which a specific compound was detected is provided as well. Concentrations (ng/ml) are rounded off to one decimal, the ratios to two decimals. 5-HIAA, 5-hydroxyindoleacetic acid; 5-HT, serotonin; A, adrenaline; DA, dopamine; DOPAC, 3-4-dihydroxyphenylacetic acid; HVA, homovanillic acid; MHPG, 3-methoxy-4-hydroxyphenylglycol; NA, noradrenaline.

**Table S6: Concentrations of monoaminergic compounds and ratios in young WT_Dp1Tyb_ mice**

|  |  | **(nor)adrenergic system** | | | | **dopaminergic system** | | | | | **serotonergic system** | | | | |
| --- | --- | --- | --- | --- | --- | --- | --- | --- | --- | --- | --- | --- | --- | --- | --- |
|  |  | **NA**  **(ng/ml)** | **A**  **(ng/ml)** | **MHPG**  **(ng/ml)** | **MHPG**  **/NA** | **DA**  **(ng/ml)** | **DOPAC**  **(ng/ml)** | **HVA**  **(ng/ml)** | **DOPAC**  **/DA** | **HVA**  **/DA** | **TRP**  **(ng/ml)** | **5-HT**  **(ng/ml)** | **5-HIAA**  **(ng/ml)** | **5HIAA**  **/5HT** | **HVA**  **/5HIAA** |
| Frontal cortex | 25% | 516.7 | 9.9 | 49.8 | 0.10 | 31.4 | 24.4 | 78.4 | 0.38 | 1.49 | 2794.3 | 349.7 | 157.7 | 0.38 | 0.45 |
|  | 50% | 550.9 | 10.9 | 70.2 | 0.15 | 47.5 | 29.0 | 98.7 | 0.67 | 2.08 | 3297.3 | 410.4 | 213.7 | 0.53 | 0.45 |
|  | 75% | 593.9 | 23.5 | 119.7 | 0.20 | 66.1 | 34.5 | 111.1 | 1.01 | 2.83 | 6218.7 | 503.5 | 247.2 | 0.61 | 0.47 |
|  | *N* | *9* | *9* | *9* | *9* | *9* | *9* | *9* | *9* | *9* | *9* | *9* | *9* | *9* | *9* |
| Temporal cortex | 25% | 429.8 | 12.9 | 87.4 | 0.19 | 103.8 | 43.5 | 99.3 | 0.38 | 0.58 | 3043.9 | 385.0 | 229.0 | 0.52 | 0.38 |
|  | 50% | 462.0 | 34.1 | 136.0 | 0.29 | 163.4 | 69.2 | 144.5 | 0.41 | 0.80 | 3740.2 | 416.2 | 242.4 | 0.58 | 0.61 |
|  | 75% | 553.9 | 43.5 | 162.3 | 0.33 | 239.3 | 85.9 | 196.0 | 0.58 | 1.57 | 6075.5 | 493.1 | 281.9 | 0.67 | 0.78 |
|  | *N* | *9* | *9* | *9* | *9* | *9* | *9* | *9* | *9* | *9* | *9* | *9* | *9* | *9* | *9* |
| Parietal cortex | 25% | 606.0 | 7.5 | 69.2 | 0.11 | 37.8 | 12.1 | 42.4 | 0.29 | 1.00 | 3144.8 | 334.5 | 206.4 | 0.46 | 0.18 |
|  | 50% | 637.2 | 16.8 | 81.3 | 0.12 | 42.6 | 19.3 | 53.4 | 0.50 | 1.36 | 3830.3 | 386.2 | 231.1 | 0.60 | 0.23 |
|  | 75% | 680.8 | 20.1 | 91.6 | 0.14 | 50.0 | 29.1 | 67.6 | 0.62 | 1.47 | 6222.6 | 446.8 | 255.7 | 0.77 | 0.35 |
|  | *N* | *9* | *8* | *9* | *9* | *9* | *9* | *9* | *9* | *9* | *9* | *9* | *9* | *9* | *9* |
| Occipital cortex | 25% | 416.2 | 8.6 | 64.8 | 0.15 | 18.5 | 4.9 | 9.4 | 0.09 | 0.32 | 3471.6 | 189.3 | 152.3 | 0.61 | 0.06 |
|  | 50% | 439.4 | 12.7 | 80.4 | 0.17 | 21.5 | 6.7 | 11.7 | 0.25 | 0.53 | 3654.0 | 224.7 | 167.6 | 0.82 | 0.06 |
|  | 75% | 461.4 | 16.3 | 86.1 | 0.20 | 49.5 | 11.5 | 15.4 | 0.50 | 0.63 | 5726.3 | 232.4 | 188.3 | 0.99 | 0.09 |
|  | *N* | *9* | *9* | *9* | *9* | *9* | *9* | *9* | *9* | *9* | *9* | *9* | *9* | *9* | *9* |
| Hippocampus | 25% | 439.6 | 5.6 | 40.2 | 0.09 | 42.6 | 16.1 | 58.6 | 0.37 | 1.16 | 3285.8 | 457.5 | 444.2 | 0.95 | 0.12 |
|  | 50% | 507.6 | 5.8 | 47.8 | 0.10 | 52.2 | 20.8 | 67.6 | 0.45 | 1.39 | 3558.0 | 476.1 | 505.5 | 1.08 | 0.13 |
|  | 75% | 550.0 | - | 66.9 | 0.12 | 57.1 | 25.0 | 84.6 | 0.48 | 1.61 | 6394.7 | 515.9 | 661.3 | 1.30 | 0.14 |
|  | *N* | *9* | *3* | *9* | *9* | *9* | *9* | *9* | *9* | *9* | *9* | *9* | *9* | *9* | *9* |
| Striatum | 25% | 155.4 | 3.9 | 55.1 | 0.27 | 9799.5 | 1333.3 | 1484.3 | 0.12 | 0.14 | 3558.4 | 404.0 | 395.2 | 1.00 | 3.33 |
|  | 50% | 203.6 | 8.3 | 67.5 | 0.33 | 10938.3 | 1476.9 | 1629.0 | 0.13 | 0.15 | 4089.3 | 415.0 | 447.9 | 1.08 | 3.63 |
|  | 75% | 236.7 | 16.6 | 78.6 | 0.42 | 12737.7 | 1653.8 | 2004.5 | 0.16 | 0.16 | 6900.1 | 433.6 | 581.4 | 1.22 | 3.77 |
|  | *N* | *9* | *7* | *9* | *9* | *9* | *9* | *9* | *9* | *9* | *9* | *9* | *9* | *9* | *9* |
| (Hypo)thalamus | 25% | 924.6 | 10.2 | 91.1 | 0.10 | 168.6 | 99.1 | 248.8 | 0.49 | 1.22 | 3383.0 | 435.5 | 575.7 | 1.23 | 0.42 |
|  | 50% | 1037.0 | 12.0 | 117.6 | 0.12 | 200.0 | 124.6 | 283.1 | 0.55 | 1.50 | 3763.1 | 470.5 | 630.7 | 1.34 | 0.46 |
|  | 75% | 1218.2 | 22.8 | 162.9 | 0.14 | 327.0 | 178.1 | 456.9 | 0.66 | 1.59 | 6177.6 | 563.6 | 755.8 | 1.57 | 0.62 |
|  | *N* | *9* | *8* | *9* | *9* | *9* | *9* | *9* | *9* | *9* | *9* | *9* | *9* | *9* | *9* |
| Brainstem | 25% | 715.5 | 3.1 | 80.0 | 0.11 | 42.9 | 40.5 | 83.2 | 0.76 | 1.48 | 2668.6 | 413.2 | 581.1 | 1.37 | 0.13 |
|  | 50% | 798.3 | 3.1 | 102.0 | 0.13 | 60.4 | 53.6 | 117.6 | 0.88 | 1.71 | 2980.6 | 451.6 | 737.9 | 1.59 | 0.14 |
|  | 75% | 811.9 | 3.1 | 116.9 | 0.14 | 75.9 | 74.1 | 143.6 | 1.13 | 2.17 | 5288.9 | 586.7 | 956.8 | 1.78 | 0.16 |
|  | *N* | *9* | *1* | *9* | *9* | *9* | *9* | *9* | *9* | *9* | *9* | *9* | *9* | *9* | *9* |
| Cerebellum | 25% | 383.5 | 2.7 | 32.3 | 0.08 | 13.5 | 10.3 | 15.4 | 0.62 | 1.00 | 3080.8 | 84.4 | 112.6 | 1.25 | 0.11 |
|  | 50% | 409.2 | 3.6 | 47.7 | 0.12 | 14.9 | 11.0 | 17.8 | 0.74 | 1.14 | 3600.2 | 104.6 | 149.9 | 1.36 | 0.14 |
|  | 75% | 443.8 | 9.8 | 69.6 | 0.16 | 20.4 | 15.1 | 20.8 | 0.87 | 1.34 | 6258.8 | 131.9 | 156.8 | 1.61 | 0.15 |
|  | *N* | *9* | *7* | *9* | *9* | *9* | *9* | *9* | *9* | *9* | *9* | *9* | *9* | *9* | *9* |
| Olfactory bulb | 25% | 304.7 | 3.3 | 41.2 | 0.12 | 223.8 | 76.3 | 216.1 | 0.31 | 0.93 | 3364.9 | 258.7 | 178.4 | 0.63 | 1.01 |
|  | 50% | 345.6 | 5.6 | 53.7 | 0.15 | 246.2 | 83.3 | 273.1 | 0.33 | 1.02 | 3696.5 | 269.2 | 191.9 | 0.71 | 1.15 |
|  | 75% | 402.1 | 9.0 | 62.4 | 0.17 | 277.7 | 108.9 | 287.0 | 0.39 | 1.11 | 7683.2 | 352.0 | 265.2 | 0.75 | 1.41 |
|  | *N* | *9* | *6* | *9* | *9* | *9* | *9* | *9* | *9* | *9* | *9* | *9* | *9* | *9* | *9* |

Monoamines, metabolites and ratios are expressed as median (50%) with the interquartile range (25%-75%). The number of samples in which a specific compound was detected is provided as well. Concentrations (ng/ml) are rounded off to one decimal, the ratios to two decimals. 5-HIAA, 5-hydroxyindoleacetic acid; 5-HT, serotonin; A, adrenaline; DA, dopamine; DOPAC, 3-4-dihydroxyphenylacetic acid; HVA, homovanillic acid; MHPG, 3-methoxy-4-hydroxyphenylglycol; NA, noradrenaline.
